# Therapeutic Targeting of Tumor Growth and Angiogenesis with a Novel Anti-S100A4 Monoclonal Antibody

**DOI:** 10.1371/journal.pone.0072480

**Published:** 2013-09-04

**Authors:** Jose Luis Hernández, Laura Padilla, Sheila Dakhel, Toni Coll, Rosa Hervas, Jaume Adan, Marc Masa, Francesc Mitjans, Josep Maria Martinez, Silvia Coma, Laura Rodríguez, Véronique Noé, Carlos J. Ciudad, Francesc Blasco, Ramon Messeguer

**Affiliations:** 1 Biomed Division, LEITAT Technological Center, Barcelona, Spain; 2 Department of Biochemistry and Molecular Biology, School of Pharmacy, University of Barcelona, Barcelona, Spain; 3 Project Area of Biopol’H, L’Hospitalet de Llobregat, Spain; Ohio State University, United States of America

## Abstract

S100A4, a member of the S100 calcium-binding protein family secreted by tumor and stromal cells, supports tumorigenesis by stimulating angiogenesis. We demonstrated that S100A4 synergizes with vascular endothelial growth factor (VEGF), via the RAGE receptor, in promoting endothelial cell migration by increasing KDR expression and MMP-9 activity. *In vivo* overexpression of S100A4 led to a significant increase in tumor growth and vascularization in a human melanoma xenograft M21 model. Conversely, when silencing S100A4 by shRNA technology, a dramatic decrease in tumor development of the pancreatic MiaPACA-2 cell line was observed. Based on these results we developed 5C3, a neutralizing monoclonal antibody against S100A4. This antibody abolished endothelial cell migration, tumor growth and angiogenesis in immunodeficient mouse xenograft models of MiaPACA-2 and M21-S100A4 cells. It is concluded that extracellular S100A4 inhibition is an attractive approach for the treatment of human cancer.

## Introduction

Angiogenesis is a crucial multi-step process in tumor growth, disease progression, and metastasis, where an orderly activation of genes controlling proliferation, invasion, migration and survival of endothelial cells (EC) participate, forming the angiogenic cascade [1],[2].

In the last decades, the active research in this field led to the development of regulatory approvals through the blockade of pathways related to VEGF, providing an effective therapeutic demonstration of the proof of concept in certain types of cancer [3], [4], [5]. According to clinical data these therapies have not produced enduring efficacy in tumor reduction or long-term survival, due to an emergent resistance to the antiangiogenic therapy [6], [7]. However, this limitation opens a new challenge for the knowledge and identification of other main factors involved in tumor angiogenesis to develop agents targeting multiple proangiogenic pathways [8], [9].

The S100 protein family, one of the largest subfamily of EF-hand calcium binding proteins, is expressed in a cell and tissue specific manner and exerts a broad range of intracellular and extracellular functions. Its members interact with specific target proteins involved in a variety of cellular processes, such as cell cycle regulation, cell growth, differentiation, motility and invasion, thus showing a strong association with some types of cancer [10], [11]. Extracellular roles for S100 members (S100B, S100A2, S100A8, S100A9, S100A12, S100P) and for S100A4 have been reported and are closely associated with tumor invasion and metastasis [12], [13].

Intracellular S100A4 is involved in: i) the motility and the metastatic capacity of cancer cells, interacting with cytoskeletal components such as the heavy chain of non-muscle myosin; ii) cell adhesion and detachment by interaction with cadherins; iii) remodeling of the extracellular matrix (ECM) by interaction with matrix metalloproteinases (MMPs), and iv) cell proliferation through its binding and sequestration of the tumor-suppressor protein p53 [10], [14], [15].

S100A4 secreted by tumor and stromal cell (macrophages, fibroblasts, and activated lymphocytes into the tumor microenvironment) is a key player in promoting metastasis; it alters the metastatic potential of cancer cells, acting as an angiogenic factor inducing cell motility, and increasing the expression of MMPs [9], [16], [17]. Therefore, S100A4 becomes a promising target for therapeutic applications by blocking angiogenesis and tumor progression.

S100A4 overexpression is strongly associated with tumor aggressiveness and it is correlated with poor survival prognosis in many different cancer types such as invasive pancreatic, colorectal, prostate, breast, esophageal, gastric, and hepatocellular cancer among others [18], [19], [20]. These observations suggest that S100A4 is an essential mediator of metastasis and it is a useful prognostic marker in cancer. Even though many of the biological effects have been described, the mechanisms by which S100A4 exerts these effects are not completely understood.

The purpose of the present study was to investigate the cellular mechanism of action of S100A4 in EC to better understand the characteristics, function and therapeutic applicability of this protein in the angiogenic process and tumor development. We also investigated its possible cooperation with known angiogenic factors and its implication *in vivo* in tumor development. We also sought to provide the preclinical proof of principle using an anti-S100A4 neutralizing monoclonal antibody developed in our laboratory.

## Materials and Methods

### Ethical Animal Procedures

All procedures involving experimental animals were approved by the “Ethical Committee of Animal Experimentation” of the animal facility place at Science Park of Barcelona (Platform of Applied Research in Animal Laboratory). Once approved by the Institutional ethical committee, these procedures were additionally approved by the ethical committee of the Catalonian authorities according to the Catalonian and Spanish regulatory laws and guidelines governing experimental animal care: Subcutaneous tumor xenograft procedure (Permit number DMHA-6038); Mouse immunization procedure (Permit number DMHA-4132).

Along the procedures using experimental animals, there was established a continuous supervision control of the animals that evaluated the degree of suffering of the animals and if it was the case to sacrifice them according to the defined end point criteria [21]. The euthanasia applied was by CO_2_ saturated atmosphere.

### Production of Human Recombinant S100A4

To generate the S100A4 recombinant protein, a cDNA encoding the full-length sequence of human S100A4 was obtained by RT-PCR from mRNA of the HCT-116 cell line, derived from human colon adenocarcinoma.

The primers used in the PCR reaction were 5′-actcacat*atggcgtgccctctggagaaggccctggatgtg*-3′ and 5′-actcatgagc*tcatcatttcttcctgggctgcttatctgggaa*-3′. The S100A4 sequence was cloned into the *Nde*I site of the bacterial expression vector pET28a(+) (Novagen). Positive clones were selected and confirmed by DNA sequencing. This construct was transformed into *E.coli* Tuner™ (DE3) Competent Cells (Novagen), and the protein was induced with 1 mM isopropyl-D-thiogalacto-pyranoside (IPTG; Sigma) for 6 h. Then, bacteria were harvested and lysed by sonication (2 min. at 30% amplitude and 4°C with pulses of 0.5 sec.) in buffer A (100 µg/mL lysozyme, 0.5 M NaCl, 10 mM Na_2_HPO_4_.2H_2_O, 10 mM NaH_2_PO_4_.2H_2_O and 10 mM imidazole, pH 7.5). The lysate was cleared by centrifugation and filtered through a HisTrap™ Chelating affinity column (Amersham). The purity of the supernatant containing the recombinant S100A4 protein was checked by SDS-12% (w/v) polyacrylamide gel electrophoresis.

### Monoclonal Antibody Obtention

Monoclonal antibody fusion, ELISA screening and subcloning were performed using standard technologies [22]. Maintenance, expansion and scaling up of cell cultures were carried out in a humidified atmosphere (94% air and 6% CO_2_) at 37°C. Female Balb/cAnNHsd mice (Harlan) were immunized with S100A4 fusion protein according to the following protocol. Fifty micrograms of S100A4 protein in PBS was used as an emulsion with Complete Freund’s adjuvant (Sigma) for the initial subcutaneous immunization and with Incomplete Freund’s adjuvant (Sigma) for subsequent injections at days 19 and 35. Ten days after the third injection, sera were obtained and tested. At day-51 a final boost of 25 µg of S100A4 protein in PBS was given intravenously to the mouse with the highest titrated serum. Fusion was done 4 days after the last injection. Obtained mAbs were derived from one fusion of myeloma cells with spleen cells from the selected mouse at a ratio 1/10, respectively, using PEG-1500 (Roche Diagnostics) as fusion inducer. Then, cells were plated in 96 microwell dishes in medium containing HAT (Invitrogen) for hybrids selection. Hybridoma supernatants were screened for reactivity with recombinant human S100A4 by ELISA. Clone corresponding to monoclonal antibody 5C3 was selected for in vitro and in vivo analyses and subcloned by limiting dilution.

### Monoclonal Antibody Production and Purification

Ten liters of serum free supernatant from the hybridoma were obtained. After filtration, purification was made on protein A columns (MabSelect Sure™ LX; 25 ml, Amersham) using an ÄKTA purifier FPLC system. Fractions were analyzed by SDS-PAGE. Eluted antibody was concentrated and diafiltrated in PBS with Amicon® Ultra-15 centrifugal filter devices with low-binding Ultracel® membranes (30000 NMWL, Millipore). Final conditioned antibodies were quantified at 280 nm.

### Cell Culture Conditions

Human Umbilical Vein Endothelial Cells (HUVECs, Lonza) were cultured on 1% Type B gelatin from bovine skin (Sigma) in Endothelial cell Basal Medium EBM (Lonza), supplemented with hEGF, hydrocortisone, brain bovine extract and gentamicine (EGM, Lonza), and 10% FCS (Invitrogen). HUVECs were used between passages 6–9 and all experiments were carried out at 80–85% of confluence, with the same batch of cells. Myeloma P3X63Ag8.653 (ECACC) cells were cultured in RPMI 1640 (PAA) supplemented with 10% FCS (PAA; Australian origin) plus 2 mM GlutaMAX™-I (Invitrogen). Colon carcinoma HCT-116 (ECACC), colon adenocarcinoma colo205 (ECACC), breast adenocarcinoma MDAMB231 (ECACC), melanoma M21 [23] (used with permission of Dr. D. Cheresh; The Scripps Research Institute, La Jolla, CA) and pancreatic carcinoma MiaPACA-2 (ECACC) cell lines were cultured in DMEM High-glucose (PAA) supplemented with 10% FCS (Invitrogen) plus 2 mM L-glutamine. M21-S100A4 overexpressing cell line (Leitat Technological Center) and MiaPACA-2 underexpressing S100A4 cell line (Leitat Technological Center) were cultured in DMEM High-glucose (PAA) supplemented with 10% FCS (Invitrogen) and 1 mg/mL G418 disulfate salt solution (Sigma) plus 2 mM L-glutamine. All cells were cultured at 37°C in a humidified 5% CO2-atmosphere, and were consistently free of mycoplasma as evaluated by EZ-PCR mycoplasma test kit (Biological Industries).

### Development of Stable Cell Lines

M21 melanoma cells, which did not express endogenous S100A4, were transfected using Lipofectamine™2000 reagent (Invitrogen) with a control plasmid (mock vector) or a plasmid encoding S100A4 cloned into the pcDNA3.1 vector and selected for resistance to G418 (1 mg/mL). Monoclones obtained by limiting dilution were selected and used for further studies. Pancreatic MiaPACA-2 cells, which express high levels of S100A4, were transfected using FuGENE 6 Transfection Reagent (Roche) with plasmids encoding for siRNA against S100A4 cloned into pSilencer 2.1-U6 neo (Ambion) with sense (5′-gatccg-cagggacaacgaggtggac-ttcaagaga-gtccacctcgttgtccctg-ttttttggaaa-3′) and antisense (5′-agcttttccaaaaaa-cagggacaacgaggtggac-tctcttgaa-gtccacctcgttgtccctg-gc-3′) sequences corresponding to nucleotides 193 to 213 of S100A4 cDNA (numbering referred to translation initiation as +1) flanked by BamHI and HindIII restriction sites on the 5′ and 3′ ends, respectively, or with a non-related siRNA with sense (5′-gatcc-actaccgttgttataggtg-ttcaagaga-cacctataacaacggtag-ttttttggaaa-3′) and antisense (5′-agcttttccaaaaaa-ctaccgttgttataggtg-tctcttgaa-cacctataacaacggtagt-g-3′) sequences directed against a sequence with no corresponding part in the human genome (actaccgttgttataggtg), used as a control for unspecific effects of shRNA. Stable expression of both S100A4 siRNA and control siRNA were established in MiaPACA-2 cells by G418 selection (1 mg/mL) and clonal cell lines were developed by limiting dilution and used for further studies. The effects of overexpressing and underexpressing S100A4 were confirmed by RT-real time PCR and Western blot analysis.

### Western Blot Analysis

1. *Signaling* pathway. Cell lysis and WB analyses were performed as described previously [24], [25] with the following modifications: before stimulation (see conditions in corresponding Figures), HUVEC cells were maintained 4 h in EBM alone. Cells were rinsed twice with PBS and immediately lysed with ice cold Cell Lysis Buffer (150 mM NaCl, 1% IGEPAL CA630, 5 mM EDTA, 100 µg/mL PMSF, 1 mM Na3VO4, 1 mM NaF and 50 mM Tris-HCL, pH 7.4). Lysates were cleared by centrifugation and the protein concentration was quantified with the Bradford reagent (Bio-Rad). Total extracts (60 µg) for all analyses were resolved by 7.5% SDS-PAGE under reduced conditions and transferred to BioTrace™ PVDF membranes (PALL corporation). Membranes were blocked for 1 h in TBS plus 0.1% Tween-20 and 5% skimmed dried milk, incubated overnight with the primary antibody and then with the secondary antibodies for 1 h in blocking buffer, with three washes of 10 min each in TBS plus 0.1% Tween-20 after each incubation. Signals were developed using the ECL™ Western Blotting Detection Reagents (Amersham) and exposed to Hyperfilm™ ECL (Amersham). In blocking assays, cultures were pre-treated for 2 h with 10–50–200 nM of the anti-RAGE monoclonal antibody (Chemicon) before the addition of S100A4 (3 µM). When using peptide 3 (30 µM), cells were pre-incubated for 2 h with S100A4 (3 µM) before incorporated into the cell culture.
2. *S100A4 expression*. Cells were lysed and total extracts were resolved by 12% SDS-PAGE. WB analysis was performed as describe above.

The concentrations/dilutions of the antibodies were as follows: 5C3 mouse monoclonal anti-human S100A4 (Leitat Technological Center) at 1 µg/ml; mouse anti-human RAGE (Millipore), at 2 µg/ml; goat polyclonal anti-human KDR (Cell Signaling Technology), 1∶500 dilution; rabbit polyclonal anti-human phospho-KDR (Millipore), 1∶250 dilution; rabbit polyclonal anti-human Tubulin (ICN Biomedicals), 1∶5000 dilution; mouse monoclonal anti-human phospho-p44/42 MAP kinase (Thr202/Tyr204) (Cell Signaling Technology), 1∶2000 dilution; rabbit polyclonal anti-p44/p42 MAP kinase (Cell Signaling Technology), 1∶1000 dilution. Goat anti-mouse (Jackson ImmunoResearch) at 0.04 µg/mL and goat anti-rabbit (Sigma) at a 1∶25000 dilution, were used as secondary antibodies.

Quantitation of the proteins was performed by densitometric analysis referring the results to the control in the non-stimulated condition (that represents 100% of expression). All signals intensities were normalized to α-tubulin.

### Real Time-PCR

Total RNA from cells was extracted using Trizol (Life Technologies) following the manufacturer’s specifications. Quantification of RNA was conducted using a Nanodrop ND-1000 spectrophotometer. cDNA was synthesized in a 20 mL reaction mixture containing 1 mg of total RNA, 12.5 ng of random hexamers (Roche), 10 mM dithiothreitol, 20 units of RNasin (Promega), 0.5 mM each dNTP (AppliChem), 4 mL of buffer (5x), and 200 units of Moloney murine leukemia virus reverse transcriptase (RT) (Invitrogen). The reaction was incubated at 37°C for 1 h. An aliquot of this cDNA mixture was used for PCR amplification by real time. The StepOnePlus™ Real-Time methodology from PCR Systems (Applied Biosystems) was used to perform these experiments. Taqman probes (Applied Biosystems, Barcelona) were used to determine mRNA levels of S100A4 (HS00243202_M1), AGER (Hs00542584_g1), and Adenine phosphoribosyltransferase (APRT) (HS00975725_M1), where APRT was used as an endogenous control. The final volume of the reaction was 20 mL, containing 1x TaqMan Universal PCR Mastermix (Applied Biosystems), 1x TaqMan probe (Applied Biosystems), 3 mL of cDNA and MQH_2_O. PCR cycling conditions were 10 min denaturation at 95°C, followed by 40 cycles of 15 s at 95°C and 1 min at 60°C. The mRNA amount of the target gene was calculated using the ΔΔC_T_ method, where C_T_ is the threshold cycle that corresponds to the cycle where the amount of amplified mRNA reaches the threshold of fluorescence.

### NF-κB Electrophoretic Mobility Shift Assay (EMSA)

To determine NF-κB nuclear translocation, HUVEC cells were maintained for 2 h in EBM and then stimulated with S100A4 (3 µM) in EBM for 20 minutes at 37°C. In blocking assays, cultures were pre-treated for 2 h with 200 nM of the anti-RAGE monoclonal antibody (Chemicon) before the addition of S100A4. Alternatively, 30 µM of peptide 3 was pre-incubated for 2 h with S100A4 before its addition to the cell culture. Nuclear extracts were prepared and used for EMSA as previously described [26], [27]. For binding reactions, 2 µg of nuclear extract were used in 20 µL in 15 mM Tris-HCl, pH 8, containing 15 mM NaCl, 0.5 mM EDTA, 60 mM KCl, 1 mM PMSF, and 0.006% β-mercaptoethanol. The binding reaction was started by the addition of the 22-bp ds oligonucleotide 5′-AGTTGAGGGGACTTTCCCAGGC –3′ (20,000 cpm) containing the NF-κB consensus sequence (underlined), end-labelled with [γ^−32^P]-ATP (3000Ci/mmol) and T4 polynucleotide kinase [24]. The binding reaction was allowed to proceed for 1 h at room temperature. For competition experiments, excess of unlabeled NF-κB oligonucleotide (2.5X or 5X) was added to the binding reaction as specific competitor 15 minutes before the addition of the labelled probe. For supershift assays, 2 µL of specific antibodies against NF-κB protein subunits p65/p50 (Santa Cruz Biotechnology) were incubated with nuclear extracts overnight at 4°C before the addition of the labelled probe. All reaction mixtures were subjected to PAGE on 6% gel in 0.5X TBE and run for 2 h at 200 V. Gels were dried and exposed for 4 h to Europium screens. Quantification was performed using a Storm 860 phosphorImager (GE Healthcare, Life Sicences).

### Interaction between S100A4 and RAGE by Surface Plasmon Resonance (SPR)

SPR measurements were performed on a T100 Biacore system (GE Healthcare Europe GmbH, Germany) as described previously [28] with some modifications. About 1,000RU of human recombinant RAGE-Fc Chimera (R&D Systems) were immobilized as ligand on CM5 sensor chips (Amersham) using standard amine-coupling protocol. Various concentrations of human recombinant S100A4 ranging from 0.625 to 5 µM were passed over the surface of the sensor chip at a flow-rate of 15 µL/min (60 sec. contact time). After each cycle, the surface was regenerated using 50 mM NaOH. For the competition assay, 2 µM of S100A4 was incubated with various concentrations of 5C3 anti-S100A4 mAb ranging from 60 nM to 500 nM, before applying to the chip. The data were analyzed with the Biacore Evaluation Software version 1.1.

### Migration Assay

Transwell migration assays were performed as described previously [29], with the following modifications: The Transwell HTS FluoroBlok™ Multiwell Insert System with 8 µm-pores (Becton Dickinson) was used to test the activities. The upper and lower surfaces of the membranes were coated with 15 µg/mL of Type I Collagen (Upstate) to improve the cell adhesion. Cells (5×10^4^ in EBM without serum or other supplements) were plated onto the upper side of the transwell and were incubated for 4 h at 37°C. Different concentrations of S100A4 (0.3, 1 or 3 µM), alone or in combination with VEGF (1, 3 or 10 ng/mL), in EBM without supplements were simultaneously added, just after cell seeding, to the lower compartment to test their chemotactic capacity. To test the inhibitory effect of the anti-RAGE mAb (Chemicon), 0.02, 2, or 200 nM of the antibody were added to the upper chamber of the insert 2 h before the stimulus with S100A4. To check the effect of the 3 peptides homologous to three regions of RAGE, 30 µM of each one were incubated with 3 µM of S100A4 two hours before adding both S100A4 and VEGF to the lower chamber to initiate migration. To check the inhibitory effect of the 5C3 mAb, 0.25, 0.5, 1, 2 or 4 µM of the antibody were incubated 2 h with S100A4 and VEGF or VEGF alone, before adding both to the lower chamber to initiate migration. All migratory effects were analyzed after 24 h, and migrated cells were stained and counted under a light microscope at a magnification of X10. All experiments were normalized to the positive control of cells incubated with EBM complete medium that represents 100% migration. The control is the maximum of the possible migration (migration control).

### MMP Activity Assay

Gelatin zymography analysis was performed as described previously [30] with the following modifications: Before stimulation, cells were maintained 4 h in EBM without serum or other supplements. Then, S100A4 (0.3, 1 or 3 µM) in EBM was added to the culture to analyze its capacity to increase the secretion of active forms of MMPs. To test the inhibitory effect of 5C3 mAb, 1–2 µM of the antibody were incubated 1 h with S100A4 (1 µM), before adding both to the culture. After 24 h at 37°C, supernatants were resolved in a non-reducing 8% SDS-PAGE gel copolymerized with Type A gelatine from porcine skin (Sigma) at a final concentration of 1 mg/mL. After running, MMPs present in the gel were activated for 48 h, gels were stained and bands were quantified using the NIH ImageJ imaging software.

### Cytotoxic Effect of Gemcitabine and 5C3 mAb

The cytotoxic effects of Gemcitabine and the 5C3 mAb were measured by hexosaminidase activity and by bromodeoxyuridine (BrdU) incorporation assay. Briefly, MiaPACA-2 cells were plated onto 96-well cell culture dishes (5×10^3^ cells/well) in 50 µL of culture medium. Twenty-for hours later, 50 µL of medium with several concentrations of Gemcitabine alone or in combination with 5C3 (40 nM, 100 nM) was added to each well and cells were cultured for 72 h. BrdU incorporation was determined using the Cell Proliferation Biotrak ELISA System™ kit (Amersham Biosciences) according to the manufacturer’s instructions. For hexosaminidase activity analysis, cells were washed once with PBS, after discarding the culture media. Sixty microliters of substrate solution (7.5 mM 4-nitrophenyl-N-acetyl-beta-D-glucosaminide, 0.1 M sodium citrate, 0.25% Triton X-100, pH 5.0) was added to each well and dishes were incubated at 37°C for 2 h. Color was developed by adding 90 µL of developer solution (50 mM glycine, 5 mM EDTA, pH 10.4), and the absorbance at 450 nm was measured by using a Multiskan Ascent spectrophotometer (Thermo Corporation). Data analysis was performed by normalizing the results with the negative control (untreated cells) that were considered as 100% of viability. Curves were adjusted using a sigmoid dose-response (variable slope) equation, and EC50 values were obtained from the equation:
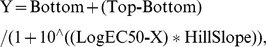
where X is the logarithm of concentration and Y is the response. Y starts at Bottom and goes to Top with a sigmoid shape.

To evaluate the level of interaction (synergistic, additive or antagonist effect) between Gemcitabine and 5C3, a variation of the method proposed by Chou-Talalay was used [31]. Briefly, the effect of the gemcitabine plus 5C3 was quantified by the combination index (CI):
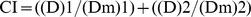
where (Dm)1 and (Dm)2 = doses of the chemicals that when applied singly also have the same effect and (D)1 and (D)2 = doses of chemicals 1 and 2 that in combination produce some specified effect.

Determining values (synergism, addition, antagonism) using the CI index as shown in Table 1:

**Table 1:** Combination index (CI) value description.

Valuerange	Description	Valuerange	Description
<0.1	Very strong synergism	0.90–1.10	Nearly additive
0.1–0.3	Strong synergism	1.20–1.45	Slight antagonism
0.3–0.7	Synergism	1.45–3.3	Antagonism
0.7–0.85	Moderate synergism	3.3–10	Strong antagonism
0.85–0.9	Slight synergism	>10	Very strong antagonism

### Tumor Growth Studies in Nude Mice

Mice for tumor models (athymic (Hsd:Athymic Nude-Foxn1nu; 6–7 weeks old)) were from Harlan Laboratories Models, S.L. (Barcelona, Spain). They were maintained within the Barcelona Science Park (PCB) animal care barrier facilities. Pancreatic MiaPACA-2 (ECACC) and melanoma M21-S100A4 (used with permission of Dr. D. Cheresh; The Scripps Research Institute, La Jolla, CA) cell lines were subcutaneously injected into the right flank of nude mice (5×10^6^ or 1×10^6^ cells for MiaPACA-2 or M21, respectively). Tumor growth was calculated using the formula: 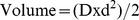, where D is the longest axis of the tumor and d is the shortest. To study the antitumorigenic capability of the 5C3 mAb, mice were treated either with vehicle (PBS) or with the antibody by i.p. route three times per week at 25 mg/Kg/5 mL of sterile PBS, starting the treatment at a mean tumor volume of approximately 100 mm^3^ for MiaPACA-2 and 115 mm^3^ for M21-S100A4 cell lines. We calculated the treatment-to-control ratio (of sample means) at the end of experiment and it corresponds to the observed RTV (Relative Tumour Volume) at a given time for the treatment and control groups, respectively. At the end of the experiment animals were sacrificed, tumors were surgically removed and weighed. Tumors were embedded in O.C.T. compound (Tissue-Tek®, Sakura) and paraffin for subsequent immunostaining analyses. Blood from all animals was collected by intracardiac puncture for posterior analyses.

### Immunohistochemical CD31 Staining

At the end of the *in vivo* experiments, subcutaneous tumors from MiaPACA-2 and M21 cells were OCT (Tissue-Tek®, Sakura) embedded and frozen. One cryosection (5 µm) corresponding to the central part of each tumor was analyzed. Sections were fixed in acetone/chloroform (1∶1) at -20°C for 5 min, dried overnight at room temperature, washed with PBS and treated for 10 min at 4°C in a dark chamber with H_2_O_2_ (0.03%) in PBS. Then, sections were washed with PBS and blocked for 20 min at 4°C using PBS-BSA (2%) plus rabbit serum (5%) (Vector) and with Avidin-biotin blocking solution (Dako) for 10 min each one at 4°C. Samples were incubated for 1 h at room temperature with primary antibody; a monoclonal rat anti-mouse antibody directed against CD31 (dil 1∶200, BD PharMingen) diluted in blocking buffer. Afterwards, sections were incubated with a polyclonal biotinylated anti-rat antibody as secondary antibody (dil 1∶500, Vector) for 30 min at room temperature and then the ABC reagent (Pierce) was added for 30 min at room temperature. Finally, sections were incubated with NovaRed (Vector) for 20 min at 4°C, stained with Haematoxylin Harris (Sigma) for 10 seconds and mounted using DPX non-aqueous mounting medium (Sigma). Angiogenesis quantification was measured using two criteria [3], [32]:
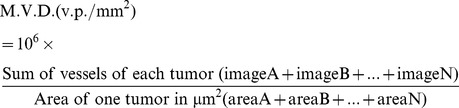


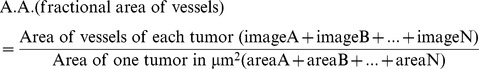



Given that the surfaces of the images taken are expressed in µm^2^ and the unit in the formula is expressed in mm^2^, a factor of 10^6^ was applied. We used individual microscopic field areas to determine the vessel density. However, in order to have a more representative value of total vasculature more than 10 pictures per slice, depending on the size of tumors, were taken and analyzed using the NIH ImageJ imaging software.

### Determination of Secreted S100A4 Protein by Sandwich ELISA Assay

To measure the presence of S100A4 in plasma samples from animals with or without tumor, a sandwich ELISA assay was performed as described previously [33] with the following modifications: Blood samples were obtained at the end of the experiments (subcutaneous tumors from HCT116, MDAMB231, MiaPACA-2 and Colo205 cell lines) by intracardiac puncture, after euthanasia. Samples for monitoring over time from animals bearing MiaPACA-2 tumors (expressing and non-expressing S100A4 protein) were collected weekly by facial puncture. All materials used were EDTA-coated. Immediately after withdrawal, blood samples were centrifuged at 5,000 rpm for 10 minutes at RT and stored at −20°C until analysis. S100A4 plasma levels were measured by a dual antibody sandwich immunoassay. 96 microtiter dishes (Maxisorb, NUNC) were coated with 10 µg/mL of 5C3 mAb diluted in PBS 24 h at 4°C. After removing the coating, dishes were washed twice with PBS and incubated 1 h at 37°C in blocking buffer (PBS containing 1% of skimmed milk). Plasma samples diluted 1∶4 in dilution buffer (PBS-4% BSA) were added to the wells and incubated 2 h at 37°C. Dishes were washed eight times with washing buffer (PBS-0.1% Tween-20) and Rabbit polyclonal anti-S100A4 secondary antibody (Dako) at 4 µg/mL was added to the wells which were incubated for 1 h at 37°C. Dishes were washed eight times with washing buffer, goat anti-rabbit-IgG-peroxidase conjugated (Sigma) at 1∶12,500 dilution was added to each well and incubated 1 h at 37°C. After washing eight times with washing buffer, the ELISA was developed by the addition of Tetramethylbenzidine substrate (Sigma) followed by incubation for 30 min at RT before stopping the reaction with 1 M of HCl. Absorbance was measured at 450 nm using a Multiskan Ascent spectrophotometer (Thermo Corporation). A standard curve was constructed by plotting absorbance values versus human S100A4 concentrations of recombinant protein (serial 1∶3 dilutions in blocking buffer starting at 8.4 µg/mL), and concentrations of unknown samples were determined using this standard curve. Correlation coefficient between plasma levels and tumor volume in subcutaneous MiaPACA-2 tumor model was obtained by linear regression. To measure the presence of secreted S100A4 in cell culture media, M21 (mock vector and S100A4 overexpressed), MiaPACA-2 (mock vector and S100A4 silenced) and HUVECs cells, were cultured in a 6-well plate with complete media until reaching 100% of confluence. Then, media were replaced for 2 mL of fresh serum free media after washing the cells twice with PBS. Supernatants were collected after 48 h and cleared by centrifugation. Presence of S100A4 protein was analyzed as we previously described for plasma samples.

### Statistical Analysis

In all studies, values are expressed as mean ± standard error of the mean (SEM) as indicated. Statistical analyses were performed by the two-tailed nonparametric Mann Whitney U test, using the GraphPad Prism software, version 5.04 for Windows. Differences were considered statistically significant at p<0.05.

## Results

### S100A4 and VEGF Exert a Synergistic Effect on HUVECs Migration, Increasing KDR Protein Expression and the Production of Active forms of MMP-9

**Figure 1:**
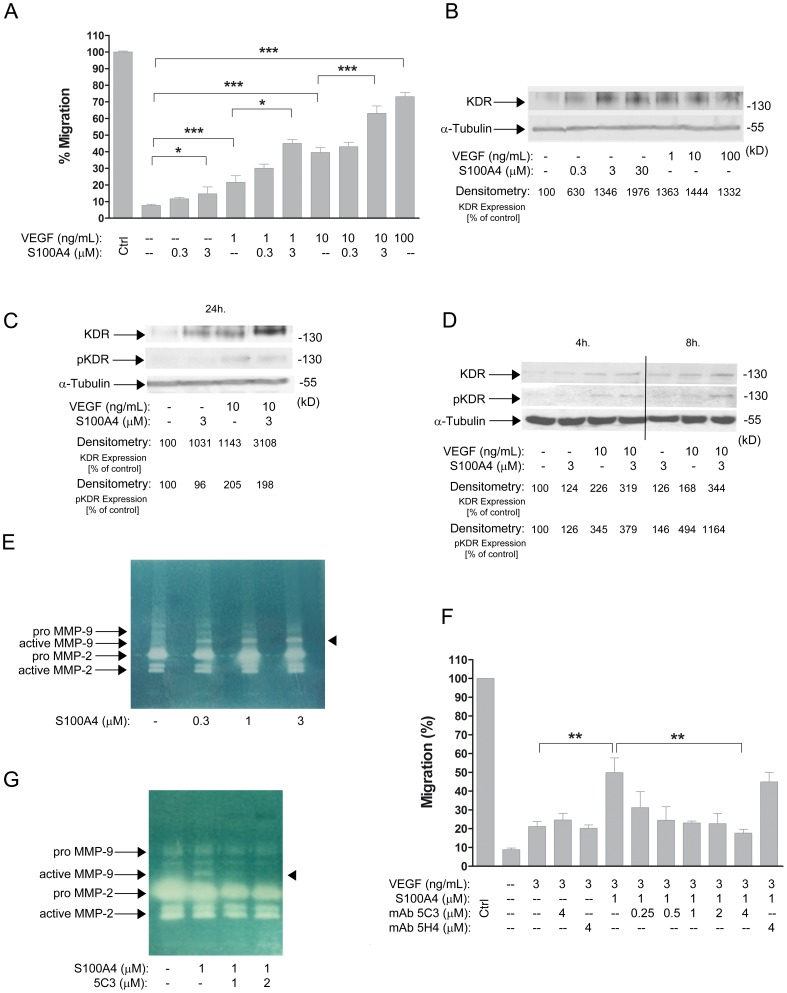
S100A4 acts synergistically with VEGF on HUVEC migration, by increasing KDR expression and the production of active MMP-9. A) Dose-response of VEGF and S100A4. Cells were treated with EBM, S100A4, VEGF, or the combination of S100A4 plus VEGF for 24 h and migration was analyzed. B) Western-blot analysis of KDR expression after adding either S100A4 (0.3–30 µM) or VEGF (1–100 ng/mL) for 24 h. C) Levels of KDR expression and activation (phosphorylation) induced by VEGF (10 ng/mL), S100A4 (3 µM) or by the combination of the two proteins for 24 h of stimulation. All KDR signal intensities were normalized to α-tubulin. D) Time course (4 h and 8 h) of KDR expression and KDR phosphorylation upon incubation with S100A4 (3 µM) or VEGF (10 ng/mL) or the combination of the two proteins. E) Proteolytic activity of MMPs in HUVEC conditioned media. Cells were treated with S100A4 in EBM alone for 24 h. Active forms of MMP-9 induced by S100A4 are indicated by arrowheads. F) HUVEC were treated with VEGF (3 ng/mL), VEGF plus S100A4 (1 µM) or the combination of these proteins with the antibody 5C3 (0.25–4 µM) for 24 h. 5H4 was used as non-blocking antibody. G) 5C3 (1–2 µM) neutralized the production of active forms of MMP-9 induced by S100A4 (arrowhead). Each data point was normalized to the positive control of cells incubated with complete medium (left bar) that represents 100% migration. The control is the maximum of the possible migration (migration control). This migration is obtained by incubating HUVEC with basal medium containing 10% FCS plus hydrocortisone, brain bovine extract and hEGF. All the experimental points are referred to this control. Bars show the mean ± SEM. ns *p*>0.05, *p<0.05, **p<0.01, ***p<0.001.

To further extend previous studies about the cellular mechanism of action of S100A4 in the angiogenic process [8], [34] we focused our work on the extracellular induced migratory capacity of this protein in HUVEC. S100A4 was tested in the dose range of 0.3–3 µM, exhibiting a small but significant migration activity at 3 µM (two-fold increased as compared to the negative control EBM). Incubation of HUVEC with 1, 10 or 100 ng/mL of VEGF alone increased migration in a dose dependent manner, by 3, 5 and 10-fold, respectively, as compared to EBM. To determine the effect of VEGF alone the migration observed by VEGF was divided by the migration obtained with the basal medium. However, when VEGF (1 or 10 ng/mL) was combined with S100A4 (0.3 or 3 µM), the effect on migration was synergistic (Fig. 1A). S100A4 and VEGF shows less migration than the control because those experimental points lack 10% FCS plus hydrocortisone, brain bovine extract and hEGF, and contains only basal medium. Therefore, this condition determines only the effect due to S100A4 and VEGF.

In particular, when 3 µM of recombinant S100A4 was added together with 1 ng/mL VEGF, migration was increased by 2.5-fold compared to the effect of VEGF alone. To obtain the 2.5 fold increase it is needed to subtract the basal migration (7% of the control) obtained in the absence of any stimulus. The combination index (Chou-Talalay method) that evaluates the level of interaction between the two proteins was 0.32, demonstrating a synergistic effect between S100A4 and VEGF. We also tested if bFGF, could exert a similar synergy together with S100A4. However, this combination was not synergic on the stimulation of HUVEC migration (data not shown).

To gain insight into one possible molecular mechanism to explain the observed synergic activity, we quantified the expression of KDR by Western Blot based on the effect observed in similar *in vitro* assays between VEGF and bFGF [35], [36], [37]. A dose-dependent effect in KDR expression was observed 24 h after S100A4 stimulation, resulting in a 6, 12 or 20-fold increase at 0.3, 3 or 30 µM S100A4, respectively, compared to the control. VEGF increased KDR expression about 12-fold, although maximum response was already observed with 1 ng/mL (Fig. 1B). Interestingly, the combination of VEGF (10 ng/mL) with S100A4 (3 µM) increased KDR protein expression by 30-fold (Fig. 1C), whereas VEGF or S100A4 alone increased only by 12-fold each, resulting in a clear synergistic effect for the combined activity of the two proteins that could explain the observed increase on migration. The concentration of 10 ng/mL VEGF was used since there was still a slight increase when compared with 1 ng/mL to be sure that we were at a steady level of VEGF to detect the increment in combination with S100A4.

In addition to KDR expression, we also checked the levels of KDR phosphorylation 24 h after stimulation with S100A4 (3 µM), VEGF (10 ng/mL) or by the combination of the two proteins. We observed that only VEGF increased KDR phosphorylation about 2-fold compared to the control at this time point (Fig. 1C). To correlate the increase in KDR expression with the activity mediated by the receptor induced by the combination of S100A4 and VEGF, a more detailed time course including 4 h and 8 h was also carried out determining both, the expression and he phosphorylation of KDR. It is observed that at 8 h of incubation with S100A4 plus VEGF there was a 2-fold increase in the phosphorylation of KDR compared with that produced by VEGF alone (Fig. 1D).

To further explore the ability of extracellular S100A4 to increase the expression and the activity of MMPs that led the cell movement and invasion [9], [38], [39] we investigated the production and secretion of activated forms of MMP-2 and MMP-9 to the conditioned medium of HUVEC after S100A4 stimulation. While no differences were observed on MMP-2 activation, a more activated form of MMP-9 was observed in cells treated with S100A4 as shown by a blanched area (arrow head in Fig. 1E), that was not detected in the medium from cells maintained in the cell culture medium alone.

Taken together, these results on EC migration, present a novel mechanism of synergistic action between VEGF and S100A4 in the angiogenic process, by overexpressing KDR and generating activated forms of MMP-9.

### 5C3 Monoclonal Antibody Blocks the Cellular Activity of S100A4 in Endothelial Cells

We next investigated the *in vitro* neutralizing activity of a novel specific mAb against murine and human S100A4, developed in our laboratory, named 5C3. Due to the small effect induced by S100A4 alone on migration, we used the combination of VEGF and S100A4 to test for the efficacy of the antibody. As the synergic effect between S100A4 and VEGF was obtained at different combinations, we decided to test intermediate concentrations, that is 3 ng/mL of VEGF and 1 µM S100A4. Treatment with 5C3 abolished in a dose-dependent manner the synergistic effect of the combination of VEGF and S100A4 on EC migration. This neutralizing activity of 5C3 was statistically significant (Fig. 1F). It is noteworthy that the antibody did not affect migration induced by the vascular endothelial growth factor alone, demonstrating that the blocking effect was exclusive of the activity of S100A4 protein. By contrast, another anti-S100A4 mAb, 5H4 (same isotype as the 5C3 mAb), did not show any neutralizing effect (Fig. 1F).

In addition, when we assessed the 5C3 impact on the potential of S100A4 to increase the production of active forms of MMP-9, we observed a total inhibition of these forms (Fig. 1G).

### S100A4 Functions via RAGE in Endothelial Cells

Previous studies revealed that several S100 family members, including S100A4, function extracellularly through their binding to RAGE [13]. Accordingly, we presumed that RAGE could be the receptor responsible for the S100A4-induced effects in EC by a mechanism of action not yet described. To test this hypothesis, we set up different experiments.

**Figure 2:**
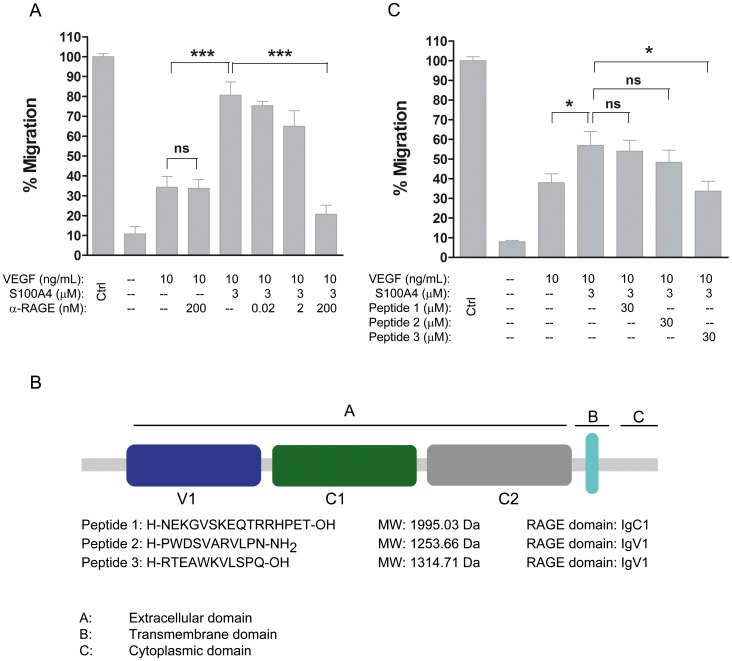
S100A4 acts via RAGE on EC migration. A) Dose-response activity of anti-RAGE (0.02–200 nM) on HUVEC migration. After starvation, cells were incubated with an anti-RAGE mAb for 2 h and then treated with VEGF (10 ng/mL) or VEGF plus S100A4 (3 µM), respectively, for 24 h. B) Design of homologous peptides to the extracellular domains of RAGE. C) Before migration, peptides were incubated with S100A4 for 2 h at 37°C, then HUVEC were treated with VEGF (10 ng/mL) or VEGF plus S100A4 (3 µM) with 30 µM of each peptide respectively for 24 h. Each data point was normalized to the positive control (left bar) that represents 100% migration. Bars show the mean ± SEM. ns *p*>0.05, *p<0.05, ***p<0.001.

As a first choice we employed an anti-RAGE mAb to inhibit the binding of S100A4 to this receptor. Using increasing concentrations of this antibody to abolish the synergistic migration effect of the combination of VEGF plus S100A4 on HUVEC, a dose-dependent inhibition was observed (Fig. 2A). The specificity of this action was also confirmed since the antibody did not affect cell migration induced by VEGF alone.

To further confirm this interaction, we designed and synthesized three peptides homologous to three different regions of the extracellular domains of the receptor to block the putative activation of RAGE by S100A4. One peptide (P1) corresponded to the extracellular domain Ig-C1, and two peptides (P2 & P3) corresponded to the extracellular domain Ig-V1. Preincubation of the peptides with S100A4 showed a maximum and statistically significant blocking effect by P3 on HUVEC migration (Fig. 2, B and C), supporting previous studies that sustain Ig-V1 as the interacting region between S100 proteins and RAGE activation [40].

**Figure 3:**
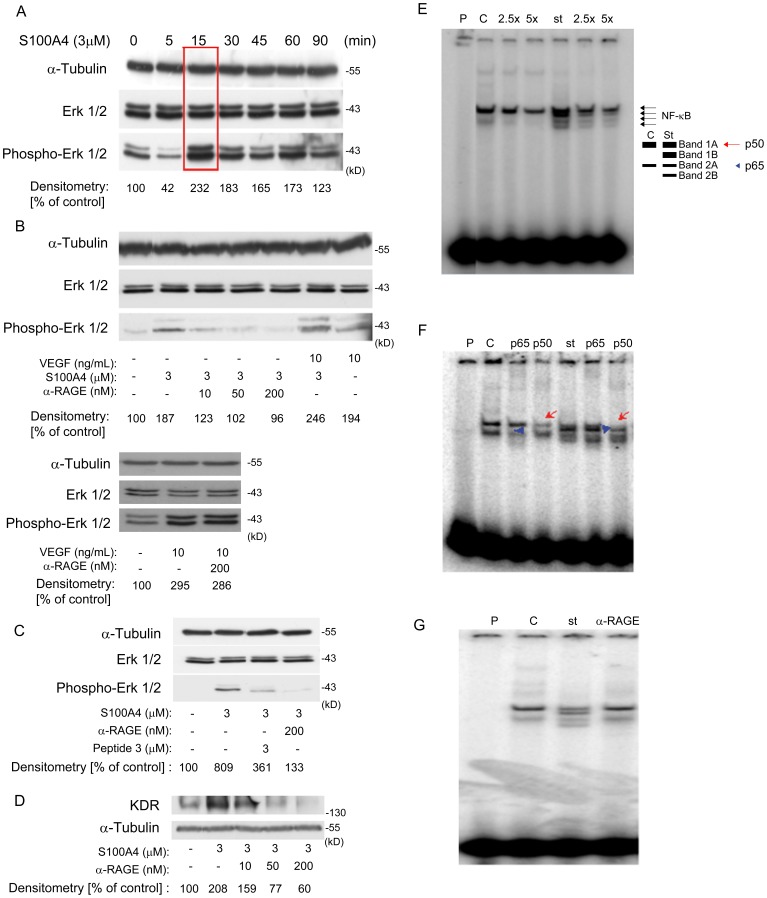
Extracellular S100A4 acts via RAGE in HUVECs, stimulating the ERK1/2, NF-κB signaling pathway and KDR expression. Cells were treated with the indicated proteins in EBM alone. Signal intensities were normalized to α-tubulin. A) Levels of ERK1/2 phosphorylation induced by 3 µM S100A4 for the indicated periods of time. B) Levels of ERK1/2 phosphorylation induced by S100A4 (3 µM), VEGF (10 ng/mL) and S100A4 plus VEGF for 15 min. Levels of ERK1/2-dependent S100A4 and VEGF phosphorylation by using the anti-RAGE mAb. C) Levels of ERK1/2 phosphorylation induced by S100A4 (3 µM) for 15 min either in the absence or in the presence of P3. D) Dose-dependent effect of anti-RAGE (10–200 nM) on S100A4-induced KDR expression. E) NF-κB binding in control cells (c) and cells treated (st) with S100A4 (3 µM) and competitions with 2.5X and 5X-fold excess of unlabelled NF-κB ds oligonucleotide. F) Supershift analysis: nuclear extracts were incubated overnight at 4°C in the presence of anti-p50 (arrow) and anti-p65 (arrowhead) antibodies before the addition of the consensus NF-κB probe. G) Gel-shift using the consensus NF-κB probe and nuclear extracts from control HUVEC (c), cells treated (st) with S100A4 (3 µM) or with the anti-RAGE antibody (200 nM), 2 h before S100A4 stimulation.

A third approach included the study of the signaling pathway downstream RAGE [41], [42]. Incubation of HUVEC with S100A4 induced ERK1/2 phosphorylation in a time-dependent manner, with a noticeable effect after 15 min (Fig. 3A). VEGF also induced ERK1/2 phosphorylation, but the combination of VEGF plus S100A4 increased the level of phosphorylation compared to the levels observed with either S100A4 or VEGF alone. ERK activation was inhibited by the addition of anti-RAGE antibody showing a dose-dependent effect or by pre-incubating S100A4 with P3 (Fig. 3B and C). The combination of VEGF and RAGE antibody did not show differences compared with VEGF alone without antibody (Fig. 3B).

In addition, we investigated whether suppression of RAGE signaling could inhibit S100A4-induced KDR expression. Using the anti-RAGE mAb a dose-dependent inhibition of the KDR protein was observed, which was completely cancelled at a concentration of 50 nM of anti-RAGE (Fig. 3D).

Incubation with S100A4 caused an increase in NF-κB binding as analyzed in HUVEC nuclear extracts. Four NF-κB bands were observed in the gel-shift assays upon S100A4 stimulation, whereas only two bands were obtained with non-stimulated cells. Binding was specific as shown by competition experiments using an excess of unlabeled ds oligonucleotide containing the NF-κB consensus binding-site (Fig. 3E). Super-shift analyses using specific antibodies for the NF-κB subunits identified the presence of p50 and p65 in the bound complexes (Fig. 3F). Activation of NF-κB binding by S100A4 was inhibited by RAGE antibody (Fig. 3G), reverting the pattern of binding to that observed with control extracts.

**Figure 4:**
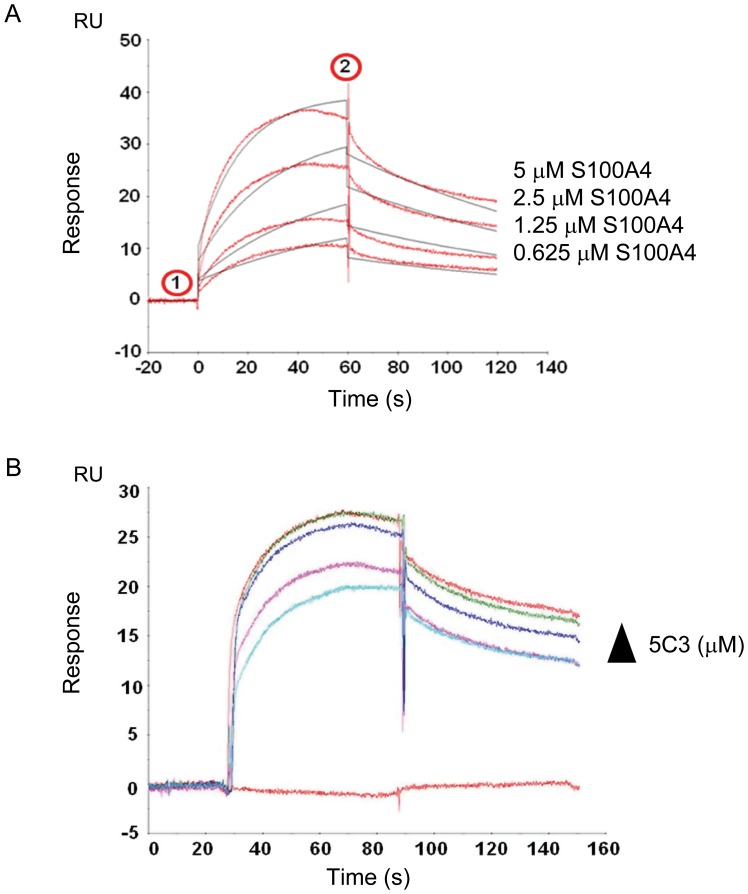
S100A4 interacts with RAGE. A) Molecular interaction between S100A4 and RAGE by SPR. Overlayed sensorgrams of interaction between immobilized RAGE and S100A4 at four different concentrations. Numbers 1 and 2 indicate the start and the end of S100A4 injection, respectively. B) Dose-response inhibition of S100A4-RAGE interaction by 5C3 mAb. Antibody was used at 60–500 nM for 2 µM S100A4.

Finally, we sought to check this interaction by surface plasmon resonance. The analysis of the sensorgram obtained in the BiaCORE after passing 0.625, 1.25, 2.5 and 5 µM of S100A4 over RAGE showed a clear dose-dependent response with the characteristic association and dissociation slopes of a true interaction between both molecules (Fig. 4A). As expected, dose-response inhibition of S100A4-RAGE interaction by the 5C3 mAb (60–125–250–500 nM) was observed with 2 µM of S100A4, confirming the neutralizing effect of the antibody against S100A4 not only at the cellular but also at the molecular level (Fig. 4B).

### Overexpression of S100A4 Promotes an Increased *in vivo* Angiogenesis and Tumor Growth

There are few data on the *in vivo* implication of extracellular S100A4 protein in tumor growth. Some authors revealed its potential role increasing tumor angiogenesis [8], [17], but so far no evidences of directed therapies against its extracellular function demonstrate its proof of principle.

According to the literature, the active extracellular form of the protein is forming oligomers whereas the intracellular is not. This conformational structure is essential for its extracellular function.

**Figure 5:**
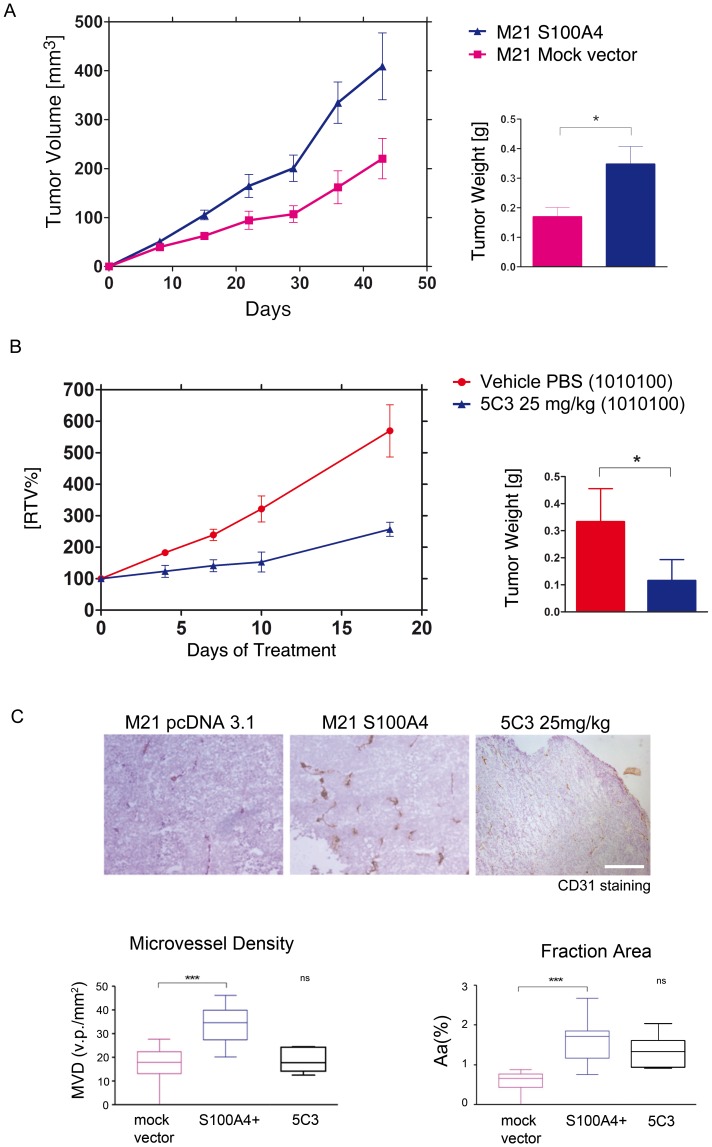
Tumorigenic study with stable S100A4 transfected cells in M21 xenograft model and effect of 5C3 on tumor growth. A) Comparison of tumor growth from M21 cells transfected either with human S100A4 or with mock vector. Groups had 15 animals. Bars of tumor weight show the mean ± SEM. *p<0.05. B) Antitumor activity of 5C3 in M21-S100A4 tumors. PBS (negative control) or 5C3 (25 mg/kg) was given i.p. three times a week (1010100) to 5 animals per group. At the end of the experiment, mice were sacrificed and tumors were weighted. Graphs of RTV show the activity of 5C3 compared with the control group. Bars of tumor weight show the mean ± SEM. *p<0.05 by. C) Immunohistology of tumor microvasculature analyzing CD31 staining. Box and whiskers graphs show the vascular density in a defined tumor area (MVD) expressed as the mean of vascular profiles (v.p.) per mm^2^, and the quantification of vessel area in the tumor (Aa). Quantifications were made from more than 10 pictures per slice at a magnification of X120 (2.4 mm^2^). Images were analyzed using the NIH ImageJ software. Graphs show the mean ± SEM. ns p>0.05, **p<0.01.

To address the question of whether S100A4 could serve as a therapeutic target *in vivo*, we evaluated the effect of S100A4 overexpression in a non-expressing human melanoma cell line (M21). After stable transfection, we selected by WB the clone with the highest expression and its function was evaluated *in vitro* and *in vivo* compared with cells transfected with the mock vector. The proliferation assay showed no differences between both cell types (data not shown), but after subcutaneous implantation in athymic mice, we observed a statistically significant increase of tumor volume when comparing S100A4-overexpressing cells to their control counterpart (Fig. 5A). Although the presence of intracellular S100A4 does not affect tumor cell proliferation we demonstrated the secretion of S100A4 in cell lines expressing this protein (Fig. S1C). This secretion affects *in vivo* the neovascular formation and therefore increases tumor growth. Keeping in mind the angiogenic role displayed by S100A4, we analyzed the formation of new microvascular vessels into the tumor in order to give a possible explanation of the dramatic observed tumor increase. Quantification of the microvessel density and the fraction area of the vessels revealed a remarkably gain of angiogenesis in tumors from animals bearing S100A4 positive cells (Fig. 5C).

To support that the observed effect was depending on the stable expression of the protein, we analyzed the presence of human S100A4 (either at mRNA or protein levels) on transfected cells (Fig. S1A and B).

To determine the possible activity of S100A4 via RAGE in these cells, we analyzed also the presence of the receptor (mRNA and protein) confirming, that effectively, all cells express RAGE without variations due to the transfection (Fig. S1A and B).

### 5C3 Decreased Tumor Growth in M21-S100A4 Overexpressing Cell Line

To further determine the role of extracellular S100A4 in increasing tumor growth, we next sought to quantify whether anti-S100A4 therapy was associated with a decreased tumor development in the M21 model. We started the treatments when solid tumors were well established (mean tumor volume 115 mm3), and continued it for 18 days. The comparison between the activity of the 5C3 antibody and the control group revealed a potent anti-tumoral effect with a decrease of 45.1% in the T/C ratio of tumor volume (Fig. 5B). Tumor weights showed differences statistically significant, with a T/C ratio of 48.1%.

In addition, we investigated the possible role of 5C3 in blocking tumor angiogenesis thus affecting tumor growth. Histological analyses of murine CD31 staining were performed for all tumor samples at the end of the experiments. Quantification of microvessel density and the fraction area of the vessels revealed an important decrease in the formed vasculature, with an inhibition about the 60% in vascular density with respect to non-treated animals (Fig. 5C).

### Knockdown of S100A4 Induced in vivo Tumor Growth Suppression

The role of S100A4 protein in *in vivo* tumor development was explored by knocking-down its expression in the human pancreatic adenocarcinoma MiaPACA-2 cell line using interfering RNA technology. Then, we determined its effect on tumor progression as in previous studies with osteosarcoma [43] or esophageal squamous carcinoma cells [44].

**Figure 6:**
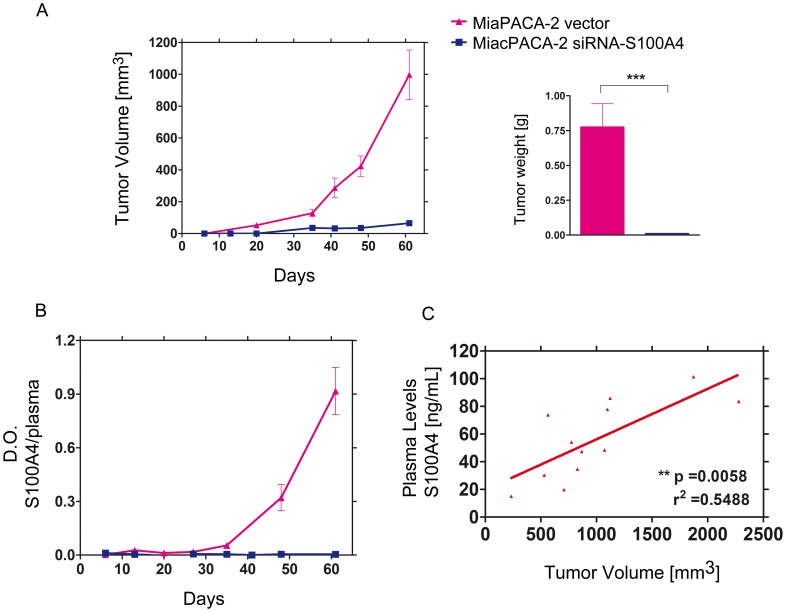
Tumorigenic study on MiaPACA-2 xenograft model silencing S100A4. A) Tumor growth of MiaPACA-2 transfected either with the pSilencer vector encoding for a shRNA against human S100A4 or for a non-related shRNA were compared. Groups had 15 animals. Graphs represent only animals that developed tumors. Bars of tumor weight show the mean ± SEM. ***p<0.001. B) S100A4 plasma levels in MiaPACA-2 transfected with shRNA-S100A4 or with a non-related shRNA were measured once a week. C) Correlation between plasma levels of S100A4 protein and tumor burden. Graphs of plasma levels show the mean ± SEM. **p<0.01. *r^2^* represents the coefficient of determination.

After stable transfection, we selected by WB the clone with the lowest S100A4 expression and its function was evaluated *in vitro* and *in vivo* by comparison with cells transfected with the mock vector. The proliferation assay showed no differences between both cell types (data not shown), but after subcutaneous implantation we observed a dramatically reduced rate of tumor growth for non-expressing S100A4 MiaPACA-2 cells; only 5 out of the 15 animals developed tiny tumors compared with the S100A4 expressing counterpart where all 15 animals developed big tumors (Fig. 6A).

We next investigated the possible correlation between the presence of S100A4 protein in plasma and tumor burden and we found an increase in animals bearing positive S100A4 tumors, following the same pattern as the growth of the corresponding tumors, whereas no S100A4 was detected in animals bearing shRNA-S100A4 cells (Fig. 6B). A statistically significant correlation was established between tumor volume and the levels of plasmatic S100A4 (Fig. 6C), and the analysis by RT-PCR and WB demonstrated the presence of mRNA and S100A4 protein, respectively, only in cells transfected with the mock vector compared with the transfected with shRNA-S100A4 (Fig. S1A and B).

To further explore the potential usefulness of the S100A4 protein as a plasmatic biomarker for diagnosis, we quantified the levels of S100A4 in plasma samples obtained from four different xenograft tumor models (MiaPACA-2, MDA-MB-231 from breast adenocarcinoma, and Colo205 and HCT116 from colon carcinoma cell lines). Experimental data demonstrated the consistent difference between the basal expression of S100A4 protein in animals without tumors and its expression in animals with tumors (Fig. S2).

### Decreased Tumor Growth in Response to Extracellular S100A4 Blockade with 5C3 in MiaPACA-2 Cell Line

To address the question of whether 5C3 mAb could serve as a therapeutic agent *in vivo* to neutralize the effect of S100A4 secreted by MiaPACA-2 cell line, we treated athymic mice bearing subcutaneous tumors.

**Figure 7:**
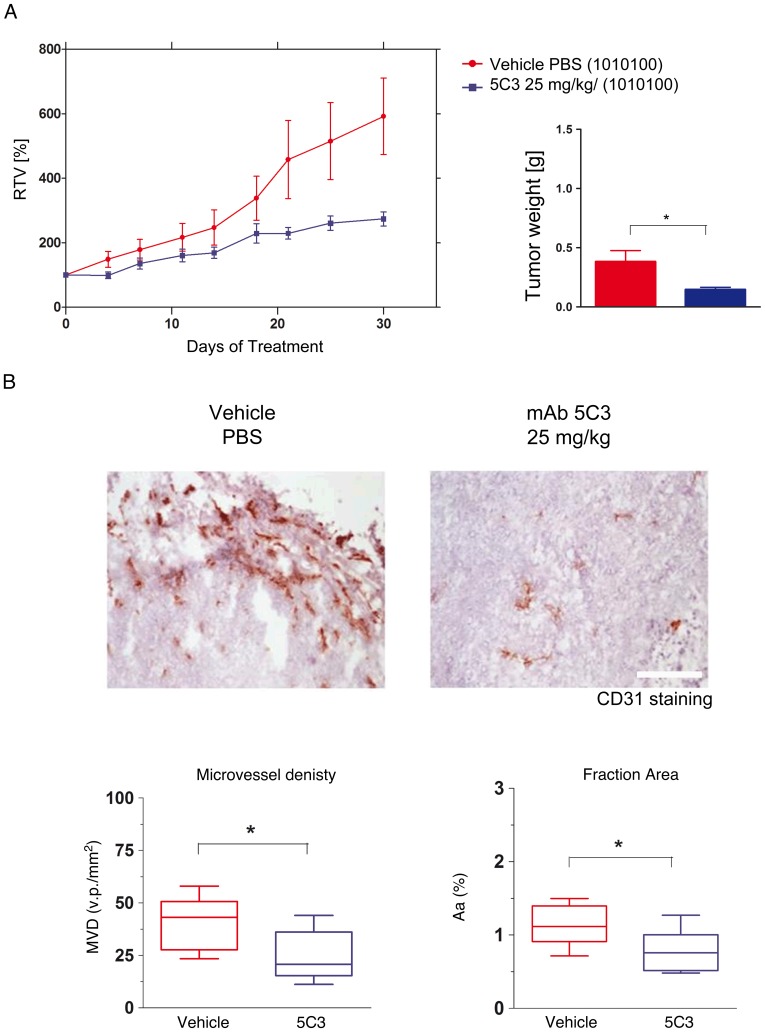
Effect of 5C3 on tumor growth in MiaPACA-2 xenograft model. A) Antitumor activity of 5C3. PBS (negative control) or 5C3 (25 mg/kg) was given i.p. three times a week (1010100). At the end of the experiment, mice were sacrificed and tumors were weighted. Graphs of RTV shows the activity of 5C3 compared with the control group. Bars of tumor weight show the mean ± SEM. *p<0.05. B) Immunohistologic analysis of tumor microvasculature from MiaPACA-2 tumors comparing PBS control group and animals treated whit 5C3. Box and whiskers graphs show the vascular density in a defined tumor area (MVD) expressed as the mean of vascular profiles (v.p.) per mm^2^, and the quantification of vessel area in the tumor (Aa). Quantifications were made from more than 10 pictures per slice at a magnification of X120 (2.4 mm^2^). Images were analyzed using the NIH ImageJ software. Graphs show the mean ± SEM. *p<0.05.

Treatment was initiated 17 days after cell implantation for MiaPACA-2 (mean tumor volume of each group higher than 100 mm^3^) (day 0), and tumors were collected at day 30 after treatment. 5C3 was given by i.p. route (25 mg/kg/5 mL), administered on a three times a week schedule. At the end of the experiment the control group (vehicle) exhibited maximum tumor growth with mean relative tumor volume (RTV) of 592% respect to the initial volume (before treatment). Tumor volume changes in 5C3-injected mice showed a maximum mean RTV of 274% respect to the initial volume. We observed that treatment with 5C3 also induced a statistically significant decrease in tumor weight compared with the control group (Fig. 7A). In addition, these differences were reflected on the calculated T/C ratios of tumor volume and tumor weight, 46%, and 38%, respectively.

We next investigated whether the 5C3 would actually affect tumor angiogenesis *in vivo* and consequently in part the tumor development. Quantification of MiaPACA-2 tumors from animals treated with the 5C3 mAb showed a statistically significant reduction of approximately 40% in microvessel density and 30% of the section area occupied by vessels compared with animals from the control group (Fig. 7B).

To gain insight into a possible combinational effect of 5C3 with conventional chemotherapy (Gemcitabine), a standard first-line treatment for advanced pancreatic cancer, we investigated the cytotoxic effect caused by the two agents in MiaPACA-2 cell line. It is noteworthy that the effect in cell viability induced by treatments with Gemcitabine alone and in combination with two concentrations of 5C3 showed a decrease in EC50 (Fig. S3A). In addition, the CI index that evaluate the level of interaction between two drugs, demonstrated a synergistic effect between the two compounds. In these experiments, proliferation was also determined. Figure S3B showed a decrease in EC50 reflecting that 5C3 had an antiproliferative effect in neutralizing the extracellular role played by S100A4 protein.

## Discussion

There is a great need for understanding the mechanisms behind the angiogenic process and the metastatic spread of tumors [2], [4]. VEGF is a key regulator of tumor angiogenesis, inducing proliferation, differentiation, and migration of EC [45]; consequently numerous drugs have been developed to target its function and its receptors [5]. Current strategies combining antiangiogenic therapy with cytotoxic agents have shown proven efficacy in cancer patients [46].

In this regard, and taken in consideration the limitations of antiangiogenic therapies, the identification of new actors, as the S100A4 protein, playing an important role not only at this stage but also in other tumor processes such as invasion, tumor inflammation, interaction between tumor cells and their microenvironment and the formation of metastatic niches, will give promising strategies for cancer therapy [10].

Recently, the role of S100A4 in tumor-associated angiogenesis as well as in EC migration has been suggested [8], [9], [47], [48] indicating that S100A4 could act in association with other angiogenic factors to achieve responses both *in vitro* and *in vivo*.

Here, we further extend previous studies about the role played by S100A4 in tumor angiogenesis and development, demonstrating that extracellular S100A4 blockade with a specific monoclonal antibody: (i) inhibits angiogenesis *in vitro* by blocking EC migration induced by the combination of S100A4 and VEGF; (ii) blocks the production of active forms of MMP-9 induced by S100A4; (iii) blocks the molecular interaction of S100A4 and the receptor RAGE; and (iv) reduces tumor angiogenesis and tumor growth *in vivo* in melanoma and pancreatic subcutaneous xenograft models, giving insights into a new strategy to treat tumors.

An important finding of this study involves the role played by S100A4 on cell motility and migration towards a stimulus, using HUVEC as a model. Our data show that exogenous addition of recombinant S100A4 increases cell migration by acting synergistically with VEGF in a dose-dependent manner and that targeting S100A4 with a specific mAb was able to inhibit this process.

In this context, we also explored the mechanism(s) by which this effect could be achieved, taking as starting point the fact that other S100 proteins (S100P, S100A12 and S100A14) stimulate cellular activities via the activation of RAGE [49], [50]. We addressed the interaction of S100A4 with RAGE through different *in vitro* approaches demonstrating that S100A4 elicited its activity by interacting with RAGE in HUVEC as it does in other cell types such as chondrocytes [51]. This conclusion is based on the following observations: first, full length RAGE interacts at the molecular level with S100A4 in a dose-dependent fashion in the BIAcore. Second, the effects of S100A4 on HUVEC migration correlate with its ability to activate the ERK 1/2 signaling pathway and the nuclear translocation of the transcription factor NF-kB, both associated to RAGE signaling. Further, Erk phosphorylation is sustained, in accordance to the literature [24]. And third, an anti-RAGE mAb or a peptide homologous to a region of the extracellular domain IgV1 of RAGE (P3) abrogate the combined migratory stimulus of S100A4 plus VEGF, as well as the steps of cell signaling associated to RAGE. Altogether these results showed that S100A4 acts through RAGE in HUVECs to promote the migratory response.

Given that bFGF acts synergistically with VEGF through an upregulation of KDR [35], [37], we checked whether S100A4 could play a similar role. Indeed, we demonstrated that S100A4 plus VEGF led to a synergistic increase in KDR protein levels in HUVEC, which provides a mechanistic explanation for the observed migration. The blockade of KDR expression with an anti-RAGE mAb provided additional evidences of the S100A4 mechanism of action.

At this point we considered that other factors could contribute to the potentiation elicited by S100A4 on VEGF-induced migration. Specifically, the ERK 1/2/NF-kB pathway has been associated with the regulation of expression of MMPs in several cell types [52], [53], [54], thus facilitating the degradation of the extracellular matrix. Our work indicates that human S100A4 increases the production and secretion of highly active forms of MMP-9, suggesting a relationship between MMPs activation and the migratory effect of S100A4. Other authors using an osteosarcoma cell line or chondrocytes observed a correlation between S100A4 and MMP activation [38], [51]. Therefore, S100A4 could participate in controlling basal membrane degradation of EC and in the destruction of the ECM to facilitate the invasion of tumor cells.

This fact opens a mechanistic explanation for extracellular S100A4 in which the increase on VEGF-induced migration in HUVEC promoted by S100A4 would rely on a combined action of an increase in KDR and the activation of MMPs through a signaling pathway initiated by RAGE.

To extend the knowledge regarding the inhibitory capacity of 5C3 mAb on the *in vitro* activity of the extracellular S100A4 protein, we chose two different steps in its signaling pathway, the molecular interaction with RAGE and the production of active forms of MMP-9. In both cases we noted a blockade of S100A4 activity, suggesting the potential therapeutic role of our antibody.

There is a growing body of evidence that S100A4, like others members of the S100 family, may play an important role in tumor angiogenesis, tumor growth and cancer metastasis [55], [56]. We further sought to determine whether S100A4 has a critical role in some animal tumor models.

Accordingly, we observed that S100A4 genetic transfer to a melanoma cell line induced a significant increase on tumor growth compared to its counterpart when cells were injected subcutaneously in athymic mice, while no differences were observed in cell proliferation. Tumor angiogenesis analysis from these cells showed a dramatic increase in vascularization, phenomenon that could explain the role of secreted S100A4 by tumor cells, therefore increasing in part the measured tumor growth. To further determine the hypothesis that this effect was due in part by the presence of extracellular S100A4, we treated animals bearing tumors from cells overexpressing S100A4 with the 5C3 monoclonal antibody, obtaining a remarkable reduction in tumor growth and tumor angiogenesis, thus indicating the importance of S100A4 on tumor development and confirming that therapies using antibodies against S100A4 can be promising strategies to treat cancer.

In this line of *in vivo* evidences, we observed that the stable silencing of S100A4 with shRNA in MiaPACA-2 cells dramatically inhibited the tumor growth. This demonstrates on the one hand the important role of S100A4 on tumor development and on the other hand that silencing is more specific than overexpression for determining the role of a factor in cell biology because the problems associated with overexpression are avoided. Moreover, the depletion of S100A4 by shRNA, which knocks down intracellular and extracellular expression, demonstrates the prominent role of the tumor cell in the crosstalk between tumor and stromal cells. Thus, these results suggest that in a therapeutic approach, it will be desirable to combine inhibitors for the intracellular and extracelluar S100A4 activity. Next we wanted to test the effectiveness of 5C3 mAb in blocking the extracellular role of S100A4 in MiaPACA-2 cells. Our findings indicated that 5C3 regressed tumor vasculature and inhibited in part tumor growth, pointing to a critical role of extracellular S100A4 during tumor progression. Then, it is possible to think of therapeutic strategies either as antiangiogenic, antitumoral or antimetastatic activities in relation to the blockade of extracellular S100A4 protein alone or in combination with other specific (e.g., anti-VEGF therapy) or generic (e.g., chemotherapy) treatments.

In order to substantiate this hypothesis, we examined 5C3′s activity in combination with the chemotherapeutic agent Gemcitabine on MiaPACA-2 cells and determined the degree of synergy. Our analysis showed a clear synergistic dose-response effect demonstrating an increase in the effectiveness of Gemcitabine treatment and furthermore opening new rationales for combined antitumor treatments.

Finally, based on the evidence that S100A4 is secreted by tumor cells and tumor activated stromal cells [57], [58], [59], and that the determination of S100A4 in plasma derived from cancer patients is feasible [60], we extended the studies and analyzed the presence of S100A4 in plasma from mice bearing tumors developed from different human cell lines. Our data further indicate that S100A4 could be considered as a good plasmatic biomarker because it allowed us to discriminate between animals with or without tumors. Moreover we can broadly affirm that 5C3 mAb is a valuable tool for use in diagnostic, and disease monitoring.

Taken all these observations together, we have elucidated a therapeutic strategy by blocking extracellular S100A4 protein with a first in class monoclonal antibody. This new drug alone or in combination with antiangiogenic or chemotherapeutic agents could be a critical inhibitory strategy to decrease tumor vasculature and consequently inhibit tumor development. Moreover, it has also been demonstrated that S100A4 can be used for monitoring treatment response and as a serum biomarker with potential diagnostic value.

A more extensive knowledge of the proteins interacting with S100A4 and the signaling pathways involved in tumor and EC, will undoubtedly be a further step in understanding the process of angiogenesis and metastasis. In the same direction, strategies designed to block any step in the signaling induced by S100A4 in tumor vasculature might represent potential approaches to tackle tumor growth and dissemination, and hence a contribution to the development of novel antitumoral and/or antiangiogenic therapies.

## Supporting Information

Figure S1 **S100A4 and RAGE expression levels.** A) Western-blot analysis of S100A4 and RAGE expression in M21 (mock vector and S100A4 overexpressed), MiaPACA-2 (mock vector and S100A4 silenced) and HUVECs cells. B) RT-PCR analysis of mRNA expression of S100A4 and RAGE. C) Secretion levels of S100A4 protein of M21, MiaPACA-2 and HUVECs cells determined by sandwich ELISA.
(TIF)

Figure S2 **S100A4 determination in plasma samples.** Plasma levels of S100A4 protein in several xenograft models in athymic mice compared with S100A4 levels in animals without tumor (no tumor) were determined by a sandwich ELISA method. One human pancreatic adenocarcinoma cell line (MiaPACA-2), two human colon adenocarcinoma cell lines (HCT116 and Colo205) and one human breast adenocarcinoma cell line (MDAMB231) were used for tumor growth. Plasma levels were measured at the end of the experiment. Graph of plasma levels shows the mean ± SEM (*n* = 10). **p<0.01, ***p<0.001.
(TIF)

Figure S3
**Cytotoxic effect of Gemcitabine combined with 5C3 mAb in MiaPACA-2 cells.** The effect of Gemcitabine, alone or in combination with 5C3 mAb, on cell viability was measured by hexosaminidase activity and BrdU incorporation. A) Dose-response effect of Gemcitabine was improved synergistically with the combination of 5C3 mAb. MiaPACA-2 cells were incubated with the chemotherapeutic drug at different doses (from 5 µM to 2 nM, dil 1∶3) with or without 5C3, at a constant concentration of 40 nM or 100 nM, for 72 h. Percentage of viability was determined in comparison to the positive control (cells without compounds) that represents 100% viability. B) Effect on proliferation for the combination of different doses of Gemcitabine (from 5 µM to 2 nM, dil 1∶3) with 5C3 at 40 nM of 100 nM, along 72 h. The level of interaction (synergistic, additive or antagonist effect) between Gemcitabine and 5C3 was quantified by the combination index (CI): 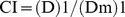 where (Dm)1 = EC50 Drug 1 concentration and (D)1 = EC50 (Drug 1+ Drug 2). The error bars represent mean ± SEM (*n* = 6).(TIF)

## References

[1] Folkman J (1971) Tumor angiogenesis: therapeutic implications. N Engl J Med 285: 1182–1186. 4938153 10.1056/NEJM197111182852108

[2] Hanahan D, Weinberg RA (2000) The hallmarks of cancer. Cell 100: 57–70. 10647931 10.1016/s0092-8674(00)81683-9

[3] Paez-Ribes M, Allen E, Hudock J, Takeda T, Okuyama H, et al (2009) Antiangiogenic therapy elicits malignant progression of tumors to increased local invasion and distant metastasis. Cancer Cell 15: 220–231. 19249680 10.1016/j.ccr.2009.01.027 PMC2874829

[4] Van der Veldt AA, Lubberink M, Bahce I, Walraven M, de Boer MP, et al (2012) Rapid decrease in delivery of chemotherapy to tumors after anti-VEGF therapy: implications for scheduling of anti-angiogenic drugs. Cancer Cell 21: 82–91. 22264790 10.1016/j.ccr.2011.11.023

[5] Ferrara N, Kerbel RS (2005) Angiogenesis as a therapeutic target. Nature 438: 967–974. 16355214 10.1038/nature04483

[6] Casanovas O, Hicklin DJ, Bergers G, Hanahan D (2005) Drug resistance by evasion of antiangiogenic targeting of VEGF signaling in late-stage pancreatic islet tumors. Cancer Cell 8: 299–309. 16226705 10.1016/j.ccr.2005.09.005

[7] Bergers G, Hanahan D (2008) Modes of resistance to anti-angiogenic therapy. Nat Rev Cancer 8: 592–603. 18650835 10.1038/nrc2442 PMC2874834

[8] Ambartsumian N, Klingelhofer J, Grigorian M, Christensen C, Kriajevska M, et al (2001) The metastasis-associated Mts1(S100A4) protein could act as an angiogenic factor. Oncogene 20: 4685–4695. 11498791 10.1038/sj.onc.1204636

[9] Schmidt-Hansen B, Ornas D, Grigorian M, Klingelhofer J, Tulchinsky E, et al (2004) Extracellular S100A4(mts1) stimulates invasive growth of mouse endothelial cells and modulates MMP-13 matrix metalloproteinase activity. Oncogene 23: 5487–5495. 15122322 10.1038/sj.onc.1207720

[10] Lukanidin E, Sleeman JP (2012) Building the niche: The role of the S100 proteins in metastatic growth. Semin Cancer Biol. 10.1016/j.semcancer.2012.02.006 22381352

[11] Heizmann CW, Ackermann GE, Galichet A (2007) Pathologies involving the S100 proteins and RAGE. Subcell Biochem 45: 93–138. 18193636 10.1007/978-1-4020-6191-2_5

[12] Boye K, Maelandsmo GM (2010) S100A4 and metastasis: a small actor playing many roles. Am J Pathol 176: 528–535. 20019188 10.2353/ajpath.2010.090526 PMC2808059

[13] Donato R (2007) RAGE: a single receptor for several ligands and different cellular responses: the case of certain S100 proteins. Curr Mol Med 7: 711–724. 18331229 10.2174/156652407783220688

[14] Mishra SK, Siddique HR, Saleem M (2011) S100A4 calcium-binding protein is key player in tumor progression and metastasis: preclinical and clinical evidence. Cancer Metastasis Rev. 10.1007/s10555-011-9338-4 22109080

[15] Garrett SC, Varney KM, Weber DJ, Bresnick AR (2006) S100A4, a mediator of metastasis. J Biol Chem 281: 677–680. 16243835 10.1074/jbc.R500017200

[16] Cabezon T, Celis JE, Skibshoj I, Klingelhofer J, Grigorian M, et al (2007) Expression of S100A4 by a variety of cell types present in the tumor microenvironment of human breast cancer. Int J Cancer 121: 1433–1444. 17565747 10.1002/ijc.22850

[17] Schmidt-Hansen B, Klingelhofer J, Grum-Schwensen B, Christensen A, Andresen S, et al (2004) Functional significance of metastasis-inducing S100A4(Mts1) in tumor-stroma interplay. J Biol Chem 279: 24498–24504. 15047714 10.1074/jbc.M400441200

[18] O’Connell JT, Sugimoto H, Cooke VG, MacDonald BA, Mehta AI, et al (2011) VEGF-A and Tenascin-C produced by S100A4+ stromal cells are important for metastatic colonization. Proc Natl Acad Sci U S A 108: 16002–16007. 21911392 10.1073/pnas.1109493108 PMC3179047

[19] Helfman DM, Kim EJ, Lukanidin E, Grigorian M (2005) The metastasis associated protein S100A4: role in tumour progression and metastasis. Br J Cancer 92: 1955–1958. 15900299 10.1038/sj.bjc.6602613 PMC2361793

[20] Sack U, Stein U (2009) Wnt up your mind - intervention strategies for S100A4-induced metastasis in colon cancer. Gen Physiol Biophys 28 Spec No Focus: F55–64. 20093727

[21] Morton DB, Griffiths PH (1985) Guidelines on the recognition of pain, distress and discomfort in experimental animals and an hypothesis for assessment. Vet Rec 116: 431–436. 3923690 10.1136/vr.116.16.431

[22] Kohler G, Milstein C (1975) Continuous cultures of fused cells secreting antibody of predefined specificity. Nature 256: 495–497. 1172191 10.1038/256495a0

[23] Cheresh DA, Spiro RC (1987) Biosynthetic and functional properties of an Arg-Gly-Asp-directed receptor involved in human melanoma cell attachment to vitronectin, fibrinogen, and von Willebrand factor. J Biol Chem 262: 17703–17711. 2447074

[24] Arumugam T, Simeone DM, Schmidt AM, Logsdon CD (2004) S100P stimulates cell proliferation and survival via receptor for activated glycation end products (RAGE). J Biol Chem 279: 5059–5065. 14617629 10.1074/jbc.M310124200

[25] Li JH, Wang W, Huang XR, Oldfield M, Schmidt AM, et al (2004) Advanced glycation end products induce tubular epithelial-myofibroblast transition through the RAGE-ERK1/2 MAP kinase signaling pathway. Am J Pathol 164: 1389–1397. 15039226 10.1016/S0002-9440(10)63225-7 PMC1615341

[26] Ciudad CJ, Morris AE, Jeng C, Chasin LA (1992) Point mutational analysis of the hamster dihydrofolate reductase minimum promoter. J Biol Chem 267: 3650–3656. 1740417

[27] Nicolas M, Noe V, Jensen KB, Ciudad CJ (2001) Cloning and characterization of the 5′-flanking region of the human transcription factor Sp1 gene. J Biol Chem 276: 22126–22132. 11294852 10.1074/jbc.M010740200

[28] Wolf S, Haase-Kohn C, Lenk J, Hoppmann S, Bergmann R, et al (2011) Expression, purification and fluorine-18 radiolabeling of recombinant S100A4: a potential probe for molecular imaging of receptor for advanced glycation endproducts in vivo? Amino Acids 41: 809–820. 21153848 10.1007/s00726-010-0822-x

[29] Keely PJ, Westwick JK, Whitehead IP, Der CJ, Parise LV (1997) Cdc42 and Rac1 induce integrin-mediated cell motility and invasiveness through PI(3)K. Nature 390: 632–636. 9403696 10.1038/37656

[30] Pazzaglia L, Ponticelli F, Magagnoli G, Gamberi G, Ragazzini P, et al (2004) Activation of metalloproteinases-2 and -9 by interleukin-1alpha in S100A4-positive liposarcoma cell line: correlation with cell invasiveness. Anticancer Res 24: 967–972. 15161051

[31] Chou TC (2008) Preclinical versus clinical drug combination studies. Leuk Lymphoma 49: 2059–2080. 19021049 10.1080/10428190802353591

[32] Mishima K, Mazar AP, Gown A, Skelly M, Ji XD, et al (2000) A peptide derived from the non-receptor-binding region of urokinase plasminogen activator inhibits glioblastoma growth and angiogenesis in vivo in combination with cisplatin. Proc Natl Acad Sci U S A 97: 8484–8489. 10890917 10.1073/pnas.150239497 PMC26974

[33] Zhu X, Munoz NM, Rubio N, Herrnreiter A, Mayer D, et al (1996) Quantitation of the cytosolic phospholipase A2 (type IV) in isolated human peripheral blood eosinophils by sandwich-ELISA. J Immunol Methods 199: 119–126. 8982353 10.1016/s0022-1759(96)00166-4

[34] Semov A, Moreno MJ, Onichtchenko A, Abulrob A, Ball M, et al (2005) Metastasis-associated protein S100A4 induces angiogenesis through interaction with Annexin II and accelerated plasmin formation. J Biol Chem 280: 20833–20841. 15788416 10.1074/jbc.M412653200

[35] Hata Y, Rook SL, Aiello LP (1999) Basic fibroblast growth factor induces expression of VEGF receptor KDR through a protein kinase C and p44/p42 mitogen-activated protein kinase-dependent pathway. Diabetes 48: 1145–1155. 10331422 10.2337/diabetes.48.5.1145

[36] Pepper MS, Mandriota SJ (1998) Regulation of vascular endothelial growth factor receptor-2 (Flk-1) expression in vascular endothelial cells. Exp Cell Res 241: 414–425. 9637783 10.1006/excr.1998.4072

[37] Stavri GT, Zachary IC, Baskerville PA, Martin JF, Erusalimsky JD (1995) Basic fibroblast growth factor upregulates the expression of vascular endothelial growth factor in vascular smooth muscle cells. Synergistic interaction with hypoxia. Circulation 92: 11–14. 7788904 10.1161/01.cir.92.1.11

[38] Bjornland K, Winberg JO, Odegaard OT, Hovig E, Loennechen T, et al (1999) S100A4 involvement in metastasis: deregulation of matrix metalloproteinases and tissue inhibitors of matrix metalloproteinases in osteosarcoma cells transfected with an anti-S100A4 ribozyme. Cancer Res 59: 4702–4708. 10493528

[39] He L, Bian L, Tang R, He Y (2011) [The invasion ability and expressions of matrix metalloproteinase-13 and calcium-binding protein S100A4 are inhibited by hyperthermia in human Tca8113 cells]. Hua Xi Kou Qiang Yi Xue Za Zhi 29: 655–659. 22332585

[40] Leclerc E, Fritz G, Vetter SW, Heizmann CW (2009) Binding of S100 proteins to RAGE: an update. Biochim Biophys Acta 1793: 993–1007. 19121341 10.1016/j.bbamcr.2008.11.016

[41] Jin Q, Chen H, Luo A, Ding F, Liu Z (2011) S100A14 stimulates cell proliferation and induces cell apoptosis at different concentrations via receptor for advanced glycation end products (RAGE). PLoS One 6: e19375. 21559403 10.1371/journal.pone.0019375 PMC3084824

[42] Grotterod I, Maelandsmo GM, Boye K (2010) Signal transduction mechanisms involved in S100A4-induced activation of the transcription factor NF-kappaB. BMC Cancer 10: 241. 20507646 10.1186/1471-2407-10-241 PMC2902441

[43] Fujiwara M, Kashima TG, Kunita A, Kii I, Komura D, et al (2011) Stable knockdown of S100A4 suppresses cell migration and metastasis of osteosarcoma. Tumour Biol 32: 611–622. 21360024 10.1007/s13277-011-0160-y

[44] Chen D, Zheng XF, Yang ZY, Liu DX, Zhang GY, et al (2012) S100A4 silencing blocks invasive ability of esophageal squamous cell carcinoma cells. World J Gastroenterol 18: 915–922. 22408350 10.3748/wjg.v18.i9.915 PMC3297050

[45] Ferrara N, Gerber HP, LeCouter J (2003) The biology of VEGF and its receptors. Nat Med 9: 669–676. 12778165 10.1038/nm0603-669

[46] Sandler A, Gray R, Perry MC, Brahmer J, Schiller JH, et al (2006) Paclitaxel-carboplatin alone or with bevacizumab for non-small-cell lung cancer. N Engl J Med 355: 2542–2550. 17167137 10.1056/NEJMoa061884

[47] Donato R (2003) Intracellular and extracellular roles of S100 proteins. Microsc Res Tech 60: 540–551. 12645002 10.1002/jemt.10296

[48] Grigorian M, Andresen S, Tulchinsky E, Kriajevska M, Carlberg C, et al (2001) Tumor suppressor p53 protein is a new target for the metastasis-associated Mts1/S100A4 protein: functional consequences of their interaction. J Biol Chem 276: 22699–22708. 11278647 10.1074/jbc.M010231200

[49] Arumugam T, Ramachandran V, Logsdon CD (2006) Effect of cromolyn on S100P interactions with RAGE and pancreatic cancer growth and invasion in mouse models. J Natl Cancer Inst 98: 1806–1818. 17179482 10.1093/jnci/djj498 PMC4461034

[50] Hofmann MA, Drury S, Fu C, Qu W, Taguchi A, et al (1999) RAGE mediates a novel proinflammatory axis: a central cell surface receptor for S100/calgranulin polypeptides. Cell 97: 889–901. 10399917 10.1016/s0092-8674(00)80801-6

[51] Yammani RR, Carlson CS, Bresnick AR, Loeser RF (2006) Increase in production of matrix metalloproteinase 13 by human articular chondrocytes due to stimulation with S100A4: Role of the receptor for advanced glycation end products. Arthritis Rheum 54: 2901–2911. 16948116 10.1002/art.22042

[52] Bond M, Chase AJ, Baker AH, Newby AC (2001) Inhibition of transcription factor NF-kappaB reduces matrix metalloproteinase-1, -3 and -9 production by vascular smooth muscle cells. Cardiovasc Res 50: 556–565. 11376631 10.1016/s0008-6363(01)00220-6

[53] Kim H, Koh G (2000) Lipopolysaccharide activates matrix metalloproteinase-2 in endothelial cells through an NF-kappaB-dependent pathway. Biochem Biophys Res Commun 269: 401–405. 10708565 10.1006/bbrc.2000.2308

[54] Huang S, Pettaway CA, Uehara H, Bucana CD, Fidler IJ (2001) Blockade of NF-kappaB activity in human prostate cancer cells is associated with suppression of angiogenesis, invasion, and metastasis. Oncogene 20: 4188–4197. 11464285 10.1038/sj.onc.1204535

[55] Salama I, Malone PS, Mihaimeed F, Jones JL (2008) A review of the S100 proteins in cancer. Eur J Surg Oncol 34: 357–364. 17566693 10.1016/j.ejso.2007.04.009

[56] McKiernan E, McDermott EW, Evoy D, Crown J, Duffy MJ (2011) The role of S100 genes in breast cancer progression. Tumour Biol 32: 441–450. 21153724 10.1007/s13277-010-0137-2

[57] Wetting HL, Hadler-Olsen E, Magnussen S, Rikardsen O, Steigen SE, et al (2011) S100A4 expression in xenograft tumors of human carcinoma cell lines is induced by the tumor microenvironment. Am J Pathol 178: 2389–2396. 21514449 10.1016/j.ajpath.2011.01.022 PMC3081199

[58] Grum-Schwensen B, Klingelhofer J, Berg CH, El-Naaman C, Grigorian M, et al (2005) Suppression of tumor development and metastasis formation in mice lacking the S100A4(mts1) gene. Cancer Res 65: 3772–3780. 15867373 10.1158/0008-5472.CAN-04-4510

[59] Olsen CJ, Moreira J, Lukanidin EM, Ambartsumian NS (2010) Human mammary fibroblasts stimulate invasion of breast cancer cells in a three-dimensional culture and increase stroma development in mouse xenografts. BMC Cancer 10: 444. 20723242 10.1186/1471-2407-10-444 PMC2933628

[60] Yang H, Zhao K, Yu Q, Wang X, Song Y, et al (2012) Evaluation of Plasma and Tissue S100A4 Protein and mRNA Levels as Potential Markers of Metastasis and Prognosis in Clear Cell Renal Cell Carcinoma. J Int Med Res 40: 475–485. 22613408 10.1177/147323001204000209

