# Supplementary figures and images for: Therapeutic Targeting of Tumor Growth and Angiogenesis with a Novel Anti-S100A4 Monoclonal Antibody
Source: PLoS One. 2013 Sep 4;8(9):e72480. doi: 10.1371/journal.pone.0072480 (PMC3762817; doi:10.1371/journal.pone.0072480)

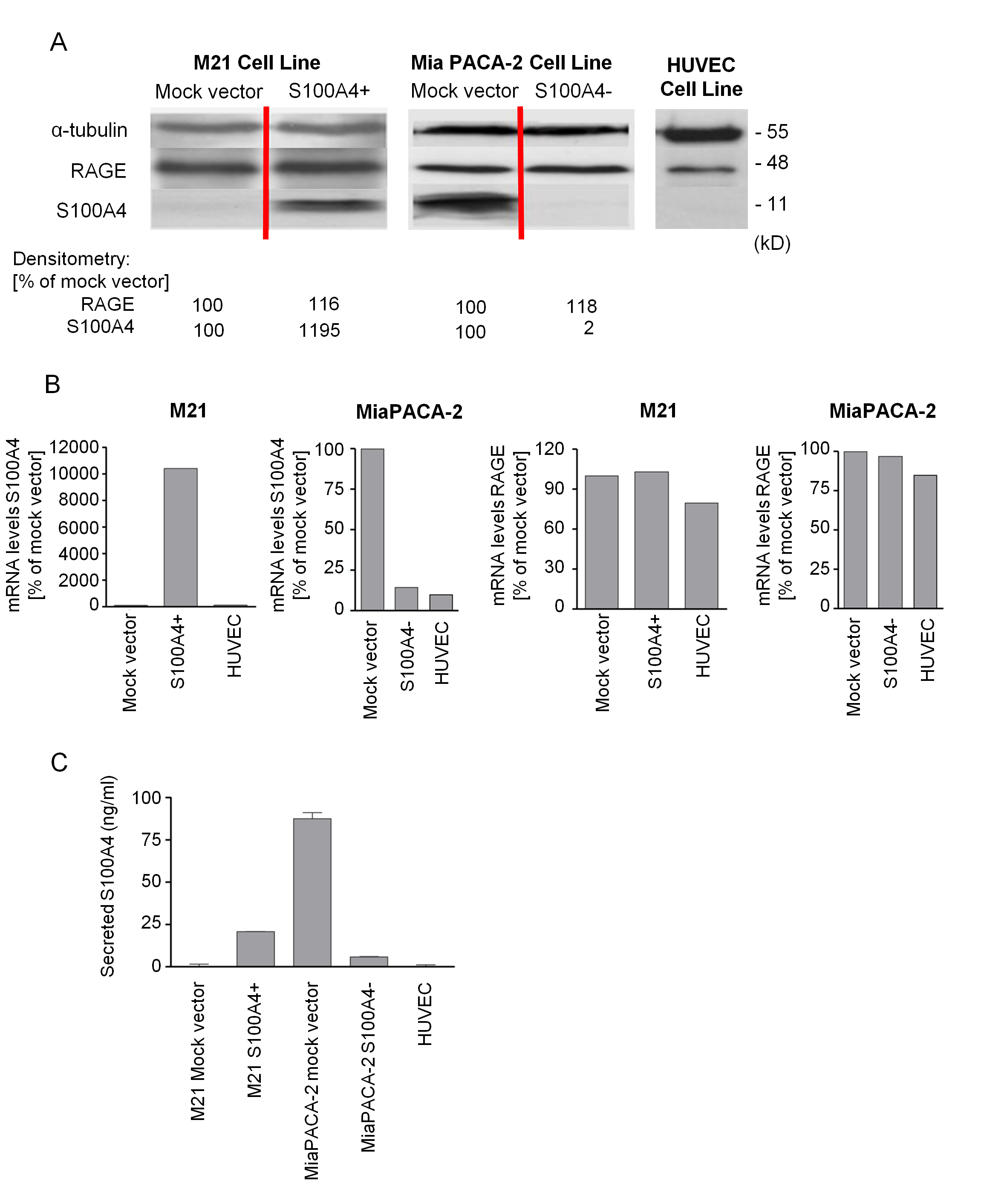

Supplement: Figure S1 — S100A4 and RAGE expression levels. A) Western-blot analysis of S100A4 and RAGE expression in M21 (mock vector and S100A4 overexpressed), MiaPACA-2 (mock vector and S100A4 silenced) and HUVECs cells. B) RT-PCR analysis of mRNA expression of S100A4 and RAGE. C) Secretion levels of S100A4 protein of M21, MiaPACA-2 and HUVECs cells determined by sandwich ELISA. (TIF) [file pone.0072480.s001.tif]

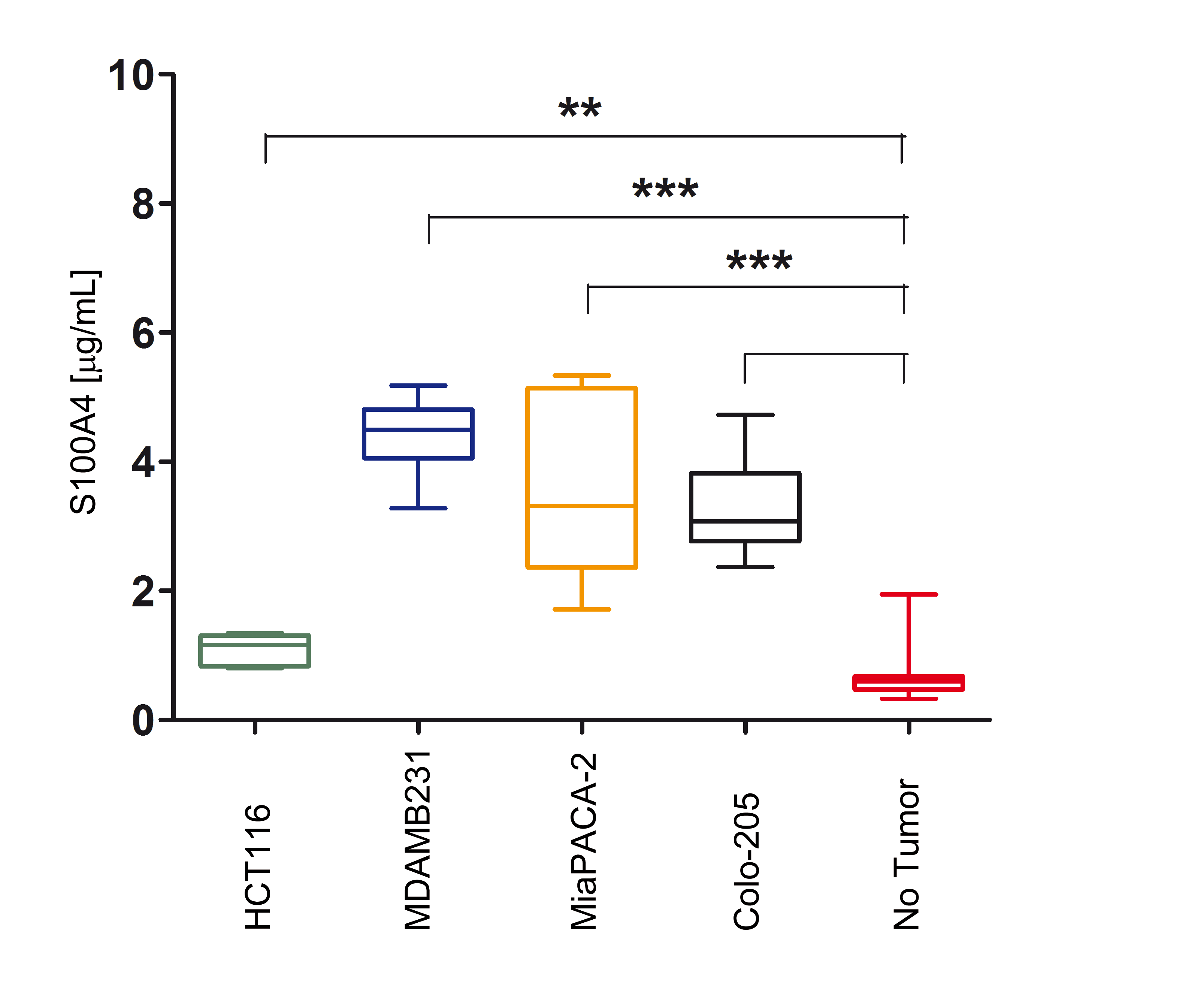

Supplement: Figure S2 — S100A4 determination in plasma samples. Plasma levels of S100A4 protein in several xenograft models in athymic mice compared with S100A4 levels in animals without tumor (no tumor) were determined by a sandwich ELISA method. One human pancreatic adenocarcinoma cell line (MiaPACA-2), two human colon adenocarcinoma cell lines (HCT116 and Colo205) and one human breast adenocarcinoma cell line (MDAMB231) were used for tumor growth. Plasma levels were measured at the end of the experiment. Graph of plasma levels shows the mean ± SEM (n = 10). **p<0.01, ***p<0.001. (TIF) [file pone.0072480.s002.tif]

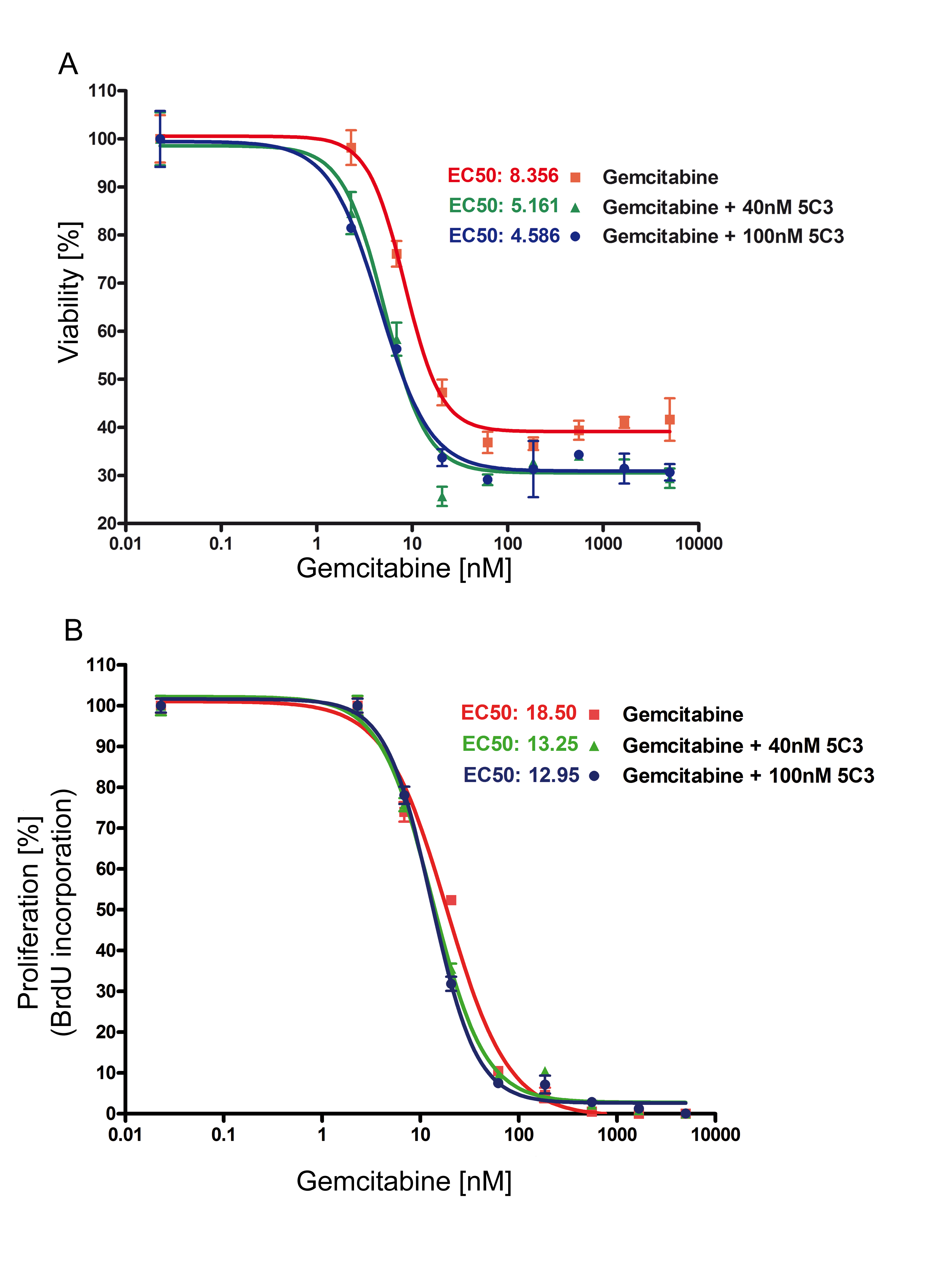

Supplement: Figure S3 — Cytotoxic effect of Gemcitabine combined with 5C3 mAb in MiaPACA-2 cells. The effect of Gemcitabine, alone or in combination with 5C3 mAb, on cell viability was measured by hexosaminidase activity and BrdU incorporation. A) Dose-response effect of Gemcitabine was improved synergistically with the combination of 5C3 mAb. MiaPACA-2 cells were incubated with the chemotherapeutic drug at different doses (from 5 µM to 2 nM, dil 1∶3) with or without 5C3, at a constant concentration of 40 nM or 100 nM, for 72 h. Percentage of viability was determined in comparison to the positive control (cells without compounds) that represents 100% viability. B) Effect on proliferation for the combination of different doses of Gemcitabine (from 5 µM to 2 nM, dil 1∶3) with 5C3 at 40 nM of 100 nM, along 72 h. The level of interaction (synergistic, additive or antagonist effect) between Gemcitabine and 5C3 was quantified by the combination index (CI): where (Dm)1 = EC50 Drug 1 concentration and (D)1 = EC50 (Drug 1+ Drug 2). The error bars represent mean ± SEM (n = 6). (TIF) [file pone.0072480.s003.tif]
